# Mastigoneme structure reveals insights into the *O*-linked glycosylation code of native hydroxyproline-rich helices

**DOI:** 10.1016/j.cell.2024.03.005

**Published:** 2024-03-28

**Authors:** Jin Dai, Meisheng Ma, Qingwei Niu, Robyn J. Eisert, Xiangli Wang, Poulomi Das, Karl F. Lechtreck, Susan K. Dutcher, Rui Zhang, Alan Brown

**Affiliations:** 1 Department of Biological Chemistry and Molecular Pharmacology, Blavatnik Institute, Harvard Medical School, Boston, MA, USA; 2 Department of Biochemistry and Molecular Biophysics, Washington University in St. Louis School of Medicine, St. Louis, MO, USA; 3 Molecular Cell Biology (MCB) graduate program, Division of Biology & Biomedical Sciences, Washington University in St. Louis School of Medicine, St. Louis, MO, USA; 4 Department of Cellular Biology, University of Georgia, Athens, GA, USA; 5 Department of Genetics, Washington University in St. Louis, St Louis, MO, USA; 6 Present address: Department of Histology and Embryology, Tongji Medical College, Huazhong University of Science and Technology, China; 7 Present address: Thermo Fisher Scientific, Shanghai, China; 8 These authors contributed equally; 9 Lead contact

## Abstract

Hydroxyproline-rich glycoproteins (HRGPs) are a ubiquitous class of protein in the extra-cellular matrices and cell walls of plants and algae, yet little is known of their native structures or interactions. Here, we used electron cryomicroscopy (cryo-EM) to determine the structure of the hydroxyproline-rich mastigoneme, an extracellular filament isolated from the cilia of the alga *Chlamydomonas reinhardtii*. The structure demonstrates that mastigonemes are formed from two HRGPs (a filament of MST1 wrapped around a single copy of MST3) that both have hyperglycosylated poly(hydroxyproline) helices. Within the helices, *O*-linked glycosylation of the hydroxyproline residues and *O*-galactosylation of interspersed serine residues create a carbohydrate casing. Analysis of the associated glycans reveals how the pattern of hydroxyproline repetition determines the type and extent of glycosylation. MST3 possesses a PKD2-like transmembrane domain that forms a heteromeric polycystin-like cation channel with PKD2 and SIP, explaining how mastigonemes are tethered to ciliary membranes.

## In brief

Structure of the native *C. reinhardtii* mastigoneme allows visualization of the glycan patterns associated with different types of proline repetition in two different hydroxyproline-rich glycoproteins, providing insight into the hydroxyproline glycosylation code. The structure also provides an explanation for how mastigonemes are tethered to ciliary membranes.

## Graphical Abstract

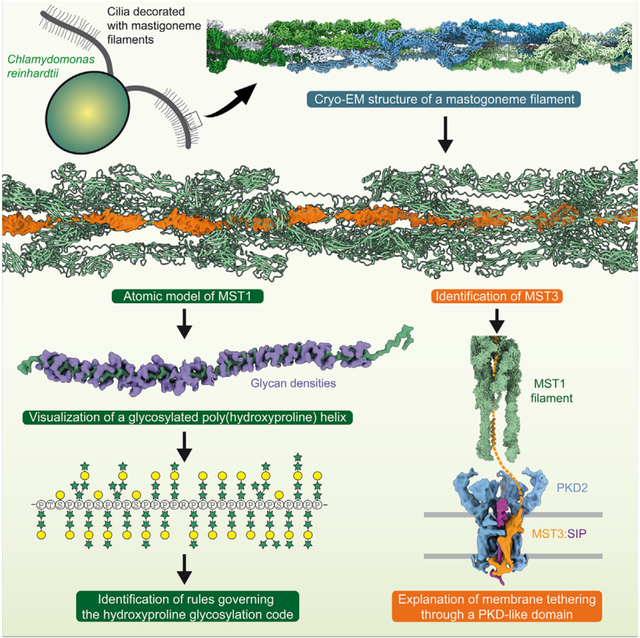

## INTRODUCTION

Poly(proline)-rich regions are common in the proteomes of many species and can form extended right- or left-handed helices depending on whether the proline residues adopt *cis* or *trans* configurations (designated as PPI and PPII, respectively).^1^ The PPII helix, in particular, is an abundant secondary structure element that adopts an extended conformation (~3.1 Å/residue) compared with classical α helices (1.5 Å/residue). Despite having no stabilizing internal hydrogen bonds, the PPII helix is thought to be relatively rigid due to the restricted range of backbone dihedral angles permitted by the pyrrolidine ring (ϕ = −75, ψ = 145°). However, our understanding of the native structures adopted by long PPII helices is limited by their paucity in the protein databank.

The proline residues within PPII helices are often post-translationally modified into hydroxyproline by prolyl 4-hydroxylases (P4H).^2^ In plants and algae, hydroxyproline residues can be further modified by extensive *O*-linked glycosylation, particularly by arabinose and galactose sugars.^3^ Hydroxyproline-rich glycoproteins (HRGPs) are ubiquitous in plant and algal cell walls,^4^ where they are thought to provide structural integrity and contribute to defense mechanisms against infection.^5^

Proline residues within PPII helices are not thought to be uniformly hydroxylated or glycosylated. For example, lysine residues block hydroxylation of adjacent prolines.^6^ Additionally, studies conducted with synthetic peptides expressed in tobacco cells have shown that the length and complexity of the glycan chains are influenced by the distribution of hydroxyprolines among other amino acids.^7,8^ These observations led to the concept of the “hydroxyproline glycosylation code”—that is, protein sequence motifs dictate the presence and extent of glycosylation.^9^ However, compared with our understanding of the *N*-glycosylation code, in which the consensus sequence N-X-S/T determines *N*-glycosylation of asparagine residues, little is known of what determines the site and scale of hydroxyproline *O*-glycosylation, the precise arrangement of these glycans, or the effect of extensive glycosylation on the structure of poly(proline) helices.^10^ The hydroxyproline glycosylation code therefore remains undecoded.

MST1, a 1,927-residue algal glycoprotein^11–13^ from the model organism *Chlamydomonas reinhardtii (C. reinhardtii)*, is notable for containing three common patterns of proline repetition within a single 78-residue poly(proline)-rich region. It has a string of nine consecutive proline residues, dyads of proline and serine, and multiple copies of the SPPP(P) motif. It is therefore a useful model for understanding the complexity of poly(proline)-rich regions found in nature.

**Figure 1.**
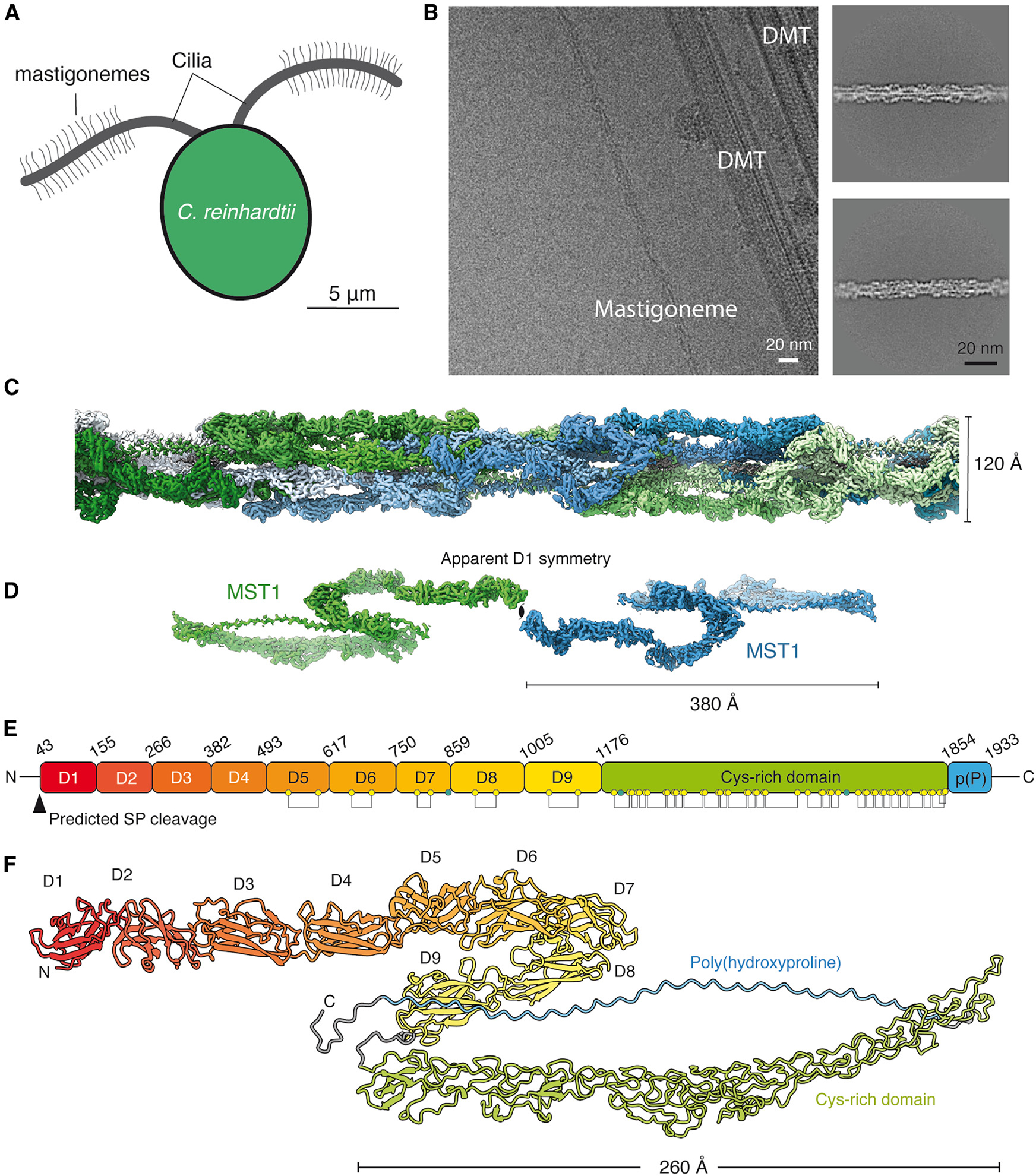
Cryo-EM structure of the *C. reinhardtii* mastigoneme (A) Schematic of *C. reinhardtii* showing mastigonemes decorating the distal two-thirds of both cilia. Mastigonemes are approximately 800 nm long. (B) Left, electron micrograph showing a mastigoneme filament and doublet microtubules (DMTs) isolated from purified C. *reinhardtii* cilia. Right, two-dimensional class averages of mastigoneme filaments. (C) Helical reconstruction of the mastigoneme filament. MST1 monomers are colored by opposing polarity and in different shades of blue and green. (D) Two MST1 monomers related by apparent D1 symmetry. (E) Domain organization of *C. reinhardtii* MST1. The first 42 residues are predicted to be a cleaved signal peptide (SP). Residues 43–1,176 form nine tandem immunoglobulin-like domains (D1–D9). Residues 1,176–1,854 form a cysteine-rich domain that contains 26 disulfide bonds (shown as connected yellow circles). Residues 1,854–1,933 form a poly(proline)-rich [p(P)] region. *N*-glycosylation sites are marked with a teal circle. (F) Atomic model of MST1 colored by domain. The C-terminal proline-rich region forms a glycosylated poly(hydroxyproline) type II helix. See Figures S1 and S2.

MST1 is proposed to be the sole constituent of mastigonemes,^11^ lateral “hairs” that fan out from the surface of cilia from some single-celled organisms^14^ (Figure 1A). The mastigonemes of *C. reinhardtii* are approximately 800 nm in length^11,15^ and taper at their distal tip into a thinner filament.^16,17^ They are asymmetrically distributed into two rows on opposite sides of the distal two-thirds of the cilium surface.^12,13^ This asymmetry is proposed to result from indirect connections between the extracellular mastigoneme and doublet microtubules 4 and 8 of the ciliary axoneme.^16^ The exact function of mastigonemes is unknown. A proposed role in increasing the efficiency of the ciliary beat^13,16,18,19^ has not been observed in all studies.^15^ A potential function in adhesion during mating has also been refuted.^12,20^ Intriguingly, recent work has shown that mastigonemes associate with a polycystin 2-like cation channel (PKD2) through an unknown mechanism in the ciliary membrane, suggesting a possible role in mechanosensation.^16^

Here, we report the structure of the native *C. reinhardtii* mastigoneme to ~3.0 Å resolution. The structure reveals that MST1 copolymerizes with a second HRGP, MST3, that likely tethers mastigonemes to the membrane through a PKD-like domain. Both proteins exhibit poly(proline) regions that form linear PPII helices, with all proline residues having been converted to *O*-glycosylated hydroxyprolines. Through examining the glycan patterns in relation to the proline sequence, we reveal general rules governing the hydroxyproline glycosylation code that may apply across both plant and algal kingdoms.

## RESULTS

### Structure determination

We found that mastigoneme filaments co-purified with *C. reinhardtii* axonemes during our work to determine structures of ciliary microtubules.^21–23^ Although mastigonemes are thought to be indirectly tethered to a subset of axonemal doublet microtubules,^16^ all the mastigonemes in our micrographs were untethered (Figures 1B and S1A), presumably having lost their connection to doublet microtubules and the ciliary membrane during treatment to dissociate the axoneme into individual microtubules. The presence of mastigoneme filaments in our micrographs (Figure 1B) presented us with an opportunity to determine their structure using single-particle helical reconstruction.

We traced each mastigoneme filament in cryoSPARC^24^ and extracted particles along their length with an arbitrary step size. A multi-step processing procedure (Figure S1 and STAR Methods) revealed helical symmetry with a rise of approximately 380 Å and a twist of 172°, creating a filament that is 12 nm at its widest and 6 nm at its thinnest (Figure 1C). The reconstruction also revealed apparent D1 point group symmetry, with its symmetry axis perpendicular to the helical axis (Figure 1D). By exploiting D1 symmetry and using masked local refinement (Figure S1A),^25^ we were able to improve the resolution of the mastigoneme to between 3.0 and 3.1 Å. The locally refined maps were aligned to a consensus map from helical refinement and stitched together to produce a map for model building.

### Structure of a MST1 monomer

The 3.0 Å resolution of the map allowed a near-complete model of MST1 to be built. Notably, due to the large size of the protein and a phylogenetic distribution limited to *Chlorophyceae* (Figure S2A), predicting the complete structure by AlphaFold2^26^ was unsuccessful (Figures S2B–S2E). However, a hybrid approach that combined de novo modeling with iterative domain-specific AlphaFold2 predictions proved successful (Figures S2F–S2H). We see no density for the first 42 residues, consistent with it being a cleaved signal peptide,^27^ or the last 37 residues.

The tertiary structure of an MST1 monomer (Figure 1F) agrees with bioinformatic analysis that it has three regions with distinct molecular identities. Residues 43–1,175, which are predicted to be rich in β-strands, form nine consecutive immunoglobulin (Ig)-like domains (D1–D9). These domains are predominantly organized as head-to-tail tandem repeats in a linear chain resembling a hook. The hook shape derives from domain pairs D5-D6 and D7-D8, which form right angles, with the second Ig-like domain of each pair interacting with the side of the preceding member. Residues 1,176–1,853, rich in cysteine residues, form a single 26-nm domain belonging to the growth factor receptor cysteine-rich domain family with 26 disulfide bonds. Residues 1,854–1,932 form a single, 22-nm long, linear PPII helix, as described in detail later.

### Mechanism of MST1 filamentation

**Figure 2.**
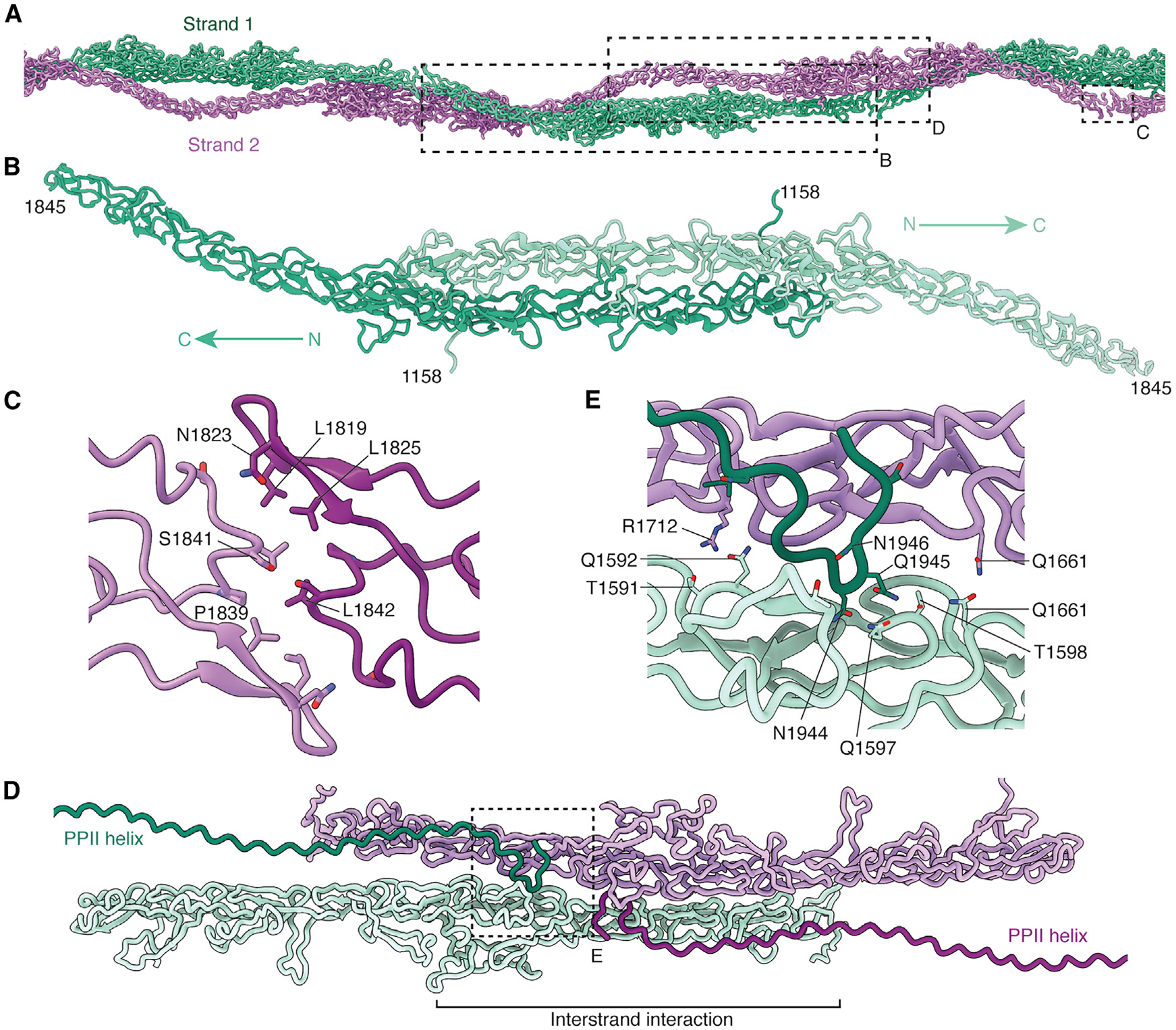
Interactions involved in the filamentation of MST1 (A) MST1 forms two non-polar strands arranged as a double helix. Repeating units of MST1 are visualized here with the immunoglobulin-like domains and the poly(proline) helix hidden to better display the strand-like architecture of the cysteine-rich domains. The boxes indicate the regions shown in (B)–(D). (B) The fundamental repeat unit of the mastigoneme is an antiparallel MST1 homodimer. Only the cysteine-rich domains are shown for clarity. (C) Intrastrand interactions between the C-terminal ends of cysteine-rich domains from adjacent homodimers. Interacting residues, which are predominantly hydrophobic, are labeled. (D) The C terminus of the poly(proline) type II (PPII) helix binds near the interstrand interface between neighboring cysteine-rich domains. The box indicates the region shown in detail in (E). (E) Details of the interactions between the C terminus of MST1 and the interstrand interface. See also Figure S1.

The mastigoneme filament can be characterized as a double helix with two non-polar MST1 polymer strands that intertwine in a spiral pattern (Figure 2A). A similar topology was identified previously at lower resolution by cryo-electron tomography.^16^ The fundamental repeat unit within each strand is an antiparallel homodimer formed through an extensive interaction between central cysteine-rich domains (Figure 2B). This interface, in excess of 7,400 Å,^2^ occurs between the N-terminal half of the cysteine-rich domain of one monomer and the C-terminal half of the same domain of the second monomer. Similar, but shorter, cysteine-rich domains mediate the dimerization of extracellular domains of growth factor receptors.^28^ The antiparallel homodimers of MST1 are polymerized through tail-to-tail interactions that involve a relatively small interface (~394 Å^2^) dominated by hydrophobic residues (Figure 2C).

As the strands coil around each other, their cysteine-rich domains interact with an interface area of ~890 Å.^2^ This interstrand interface is bound by residues that directly succeed the PPII helix (residues 1,933–1,950), creating a potentially stabilizing hydrogen-bonding network (Figures 2D and 2E). Given that this interstrand interface is larger and recognized by more elements than the tail-to-tail intrastrand interface, filamentation may precede through assembly of dimer pairs rather than the formation of two individual strands.

The nine consecutive Ig-like domains, which adorn the outside of the filament, do not seem to contribute substantially to filamentation. While they participate in some interstrand (involving D1 and D2) and intrastrand (between neighboring D6 domains) interactions, these interfaces are relatively small. Moreover, only the MST-like proteins of *C. reinhardtii* and *C. incerta* possess N-terminal Ig-like domains, suggesting that these domains are dispensable for mastigoneme filamentation in other *Chlorophyceae* species. *C. reinhardtii* also encodes an MST1 paralog (MST2; Cre06.g309900) that lacks Ig-like domains (Figure S2I), despite having cysteine- and proline-rich regions that are 53% identical. Homology modeling, based on the structure of the MST1-containing mastigoneme, suggests MST2 should also have the capability to polymerize as the interfaces in the cysteine-rich domains are structurally conserved (Figures S2J and S2K).

### The poly(proline) region forms a hydroxylated, glycosylated helix

The PPII helix, which forms from the proline-rich region near the C terminus of MST1, runs antiparallel to the cysteine-rich domain (Figure 1F). Similar to other PPII helices, it forms a left-handed, elongated structure that contains approximately three residues per turn and no internal hydrogen bonding between amino acid residues.

**Figure 3.**
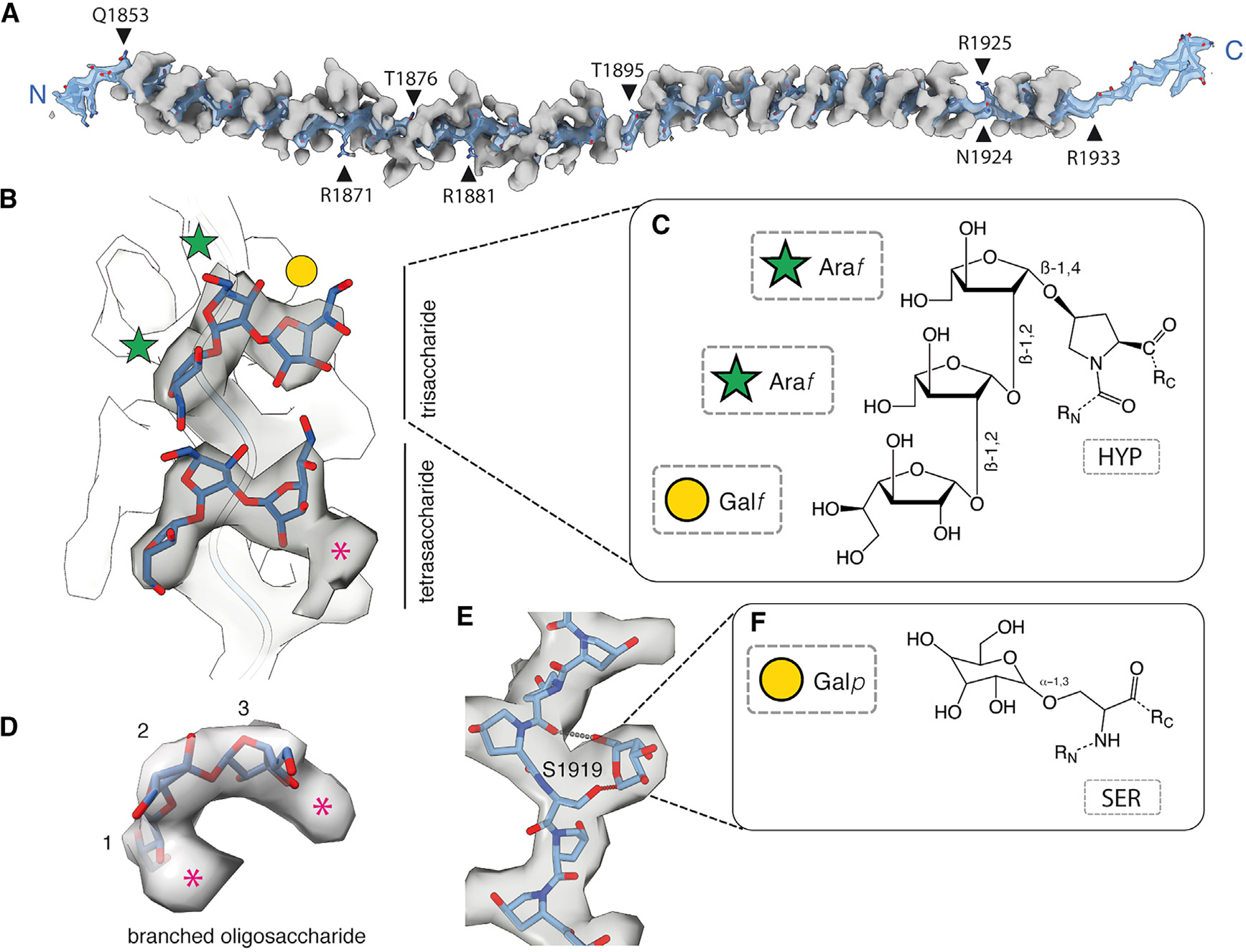
Details of the glycosylated PPII helix of MST1 (A) Cryo-EM map and atomic model of the MST1 PPII helix. The polypeptide backbone is shown as a transparent blue isosurface to reveal the atomic model. The glycans are displayed as opaque gray isosurfaces at the same contour level. Black arrowheads indicate the positions of some of the non-proline/serine residues. (B) Transparent cryo-EM map showing the density and atomic models for a tri- and tetrasaccharide bound to hydroxyproline residues within the PPII helix of MST1. Individual saccharide units are labeled according to the Symbol Nomenclature for Glycans (SNFG). An unbuilt saccharide unit in the tetrasaccharide is marked with an asterisk. (C) Proposed chemical structure of the trisaccharide glycan linked to hydroxyproline. The glycan consists of two L-arabinofuranoside moieties attached through a β1→2 glycosidic linkage to α-D-galactofuranoside (α-D-Galf). (D) An example of a branched oligosaccharide with at least five saccharide units. Additional density, marked with asterisks, supports branching after the first andthird saccharide units. (E) Cryo-EM density and atomic model for monogalactosyl serine (Ser-*O*-αGal). The monogalactosyl moiety is built in its pyranose conformation, which positions the hydroxyl group on carbon 6 such that it can hydrogen bond with the carbonyl group of the backbone of the PPII helix. Glycans associated with the neighboring hydroxyproline residues have been removed for clarity. (F) Proposed chemical structure of monogalactosyl serine. See Figure S3.

Based on the appearance of the electron cryomicroscopy (cryo-EM) density, all proline residues within the PPII helix are glycosylated (Figure 3A). We therefore modeled each glycosylated proline as *trans*-4-hydroxyproline, which is the most common hydroxyproline isomer found in nature (with a hydroxyl group on the C4 carbon atom of the proline pyrrolidine ring) and the only one known to form *O*-linked glycoconjugates.^29^ Consistent with this assignment, *C. reinhardtii* encodes multiple P4H necessary for the hydroxylation of proline,^30,31^ and *trans*-4-hydroxyproline residues are common constituents of the poly (proline) proteins of the *C. reinhardtii* cell wall.^32,33^

Close examination of the density reveals that there are a minimum of three saccharide moieties attached to each hydroxyproline residue (Figure 3B). Longer linear chains of at least four saccharide moieties (Figure 3B) and branched glycans are also present (Figure 3D). These glycans envelop the PPII helix and nestle within the polypeptide’s extended conformation (Figure 3A). This not only amplifies the caliber of the PPII helix but also encases the polypeptide backbone in a saccharide-dense shield. Consequently, the surface presented to the environment and capable of forming molecular interactions is entirely carbohydrate.

Although the identity of the glycans cannot be determined directly from the cryo-EM density, chemical and spectroscopic analyses of HRGPs of algal and plant cell walls suggest that L-Arabinose (L-Ara), a plant pentose saccharide not found in animals, is the main constituent capable of forming a glycosidic linkage to hydroxyproline.^34^ Indeed, the arabino-hydroxyproline linkage is found in all plant proteins that contain hydroxyproline and is a characteristic feature of HRGPs.^35^ In plants, this hydroxyproline-linked L-Ara is extended to form linear chains of three, four, or five L-arabinosides, with each adopting an unusual furanose conformation (L-Ara*f*).^36,37^ However, previous work^32,37,38^ demonstrates that *C. reinhardtii* is capable of a more complex pattern of *O*-linked hydroxyproline glycosylation than that found in plants. Not only can they form branched chains but the linear species can also contain non-L-Ara saccharides.^37^ In the linear chains, the first two proximal saccharides linked to hydroxyproline are the same as in plants (β-L-Araf-(1→2)-β-L-Ara*f*→HYP). These are then capped through a β1→2 glycosidic linkage by α-D-galactofuranoside (α-D-Gal*f*), which can be further methylated^37^ to create a trisaccharide (Figure 3C). An atomic model of this glycan fits the density well (Figure 3B). The conservation of the inner core between algae and plants provides confidence of α-L-arabinosides in the first and second positions linked directly to hydroxyproline.^32^

Modeling the longer linear and branched oligosaccharides presents a significant challenge due to the lack of information about native *O*-linked glycans in algal HRGPs. Some of the only information comes from an analysis of the glycans bound to GP1, a *C. reinhardtii* cell wall glycoprotein with a high percentage (37%) of branched heteroarbinosides,^39^ which revealed that up to 10 saccharide moieties are possible, with branching common for larger saccharides.^38^ Besides L-arabinofuranose and D-galactofuranose, glucose (8%), xylose (4%), and mannose (1%) were also detected.^39^ Given the array of possibilities, we opted to leave the branched glycans only partially modeled as trisaccharides.

**Figure 4.**
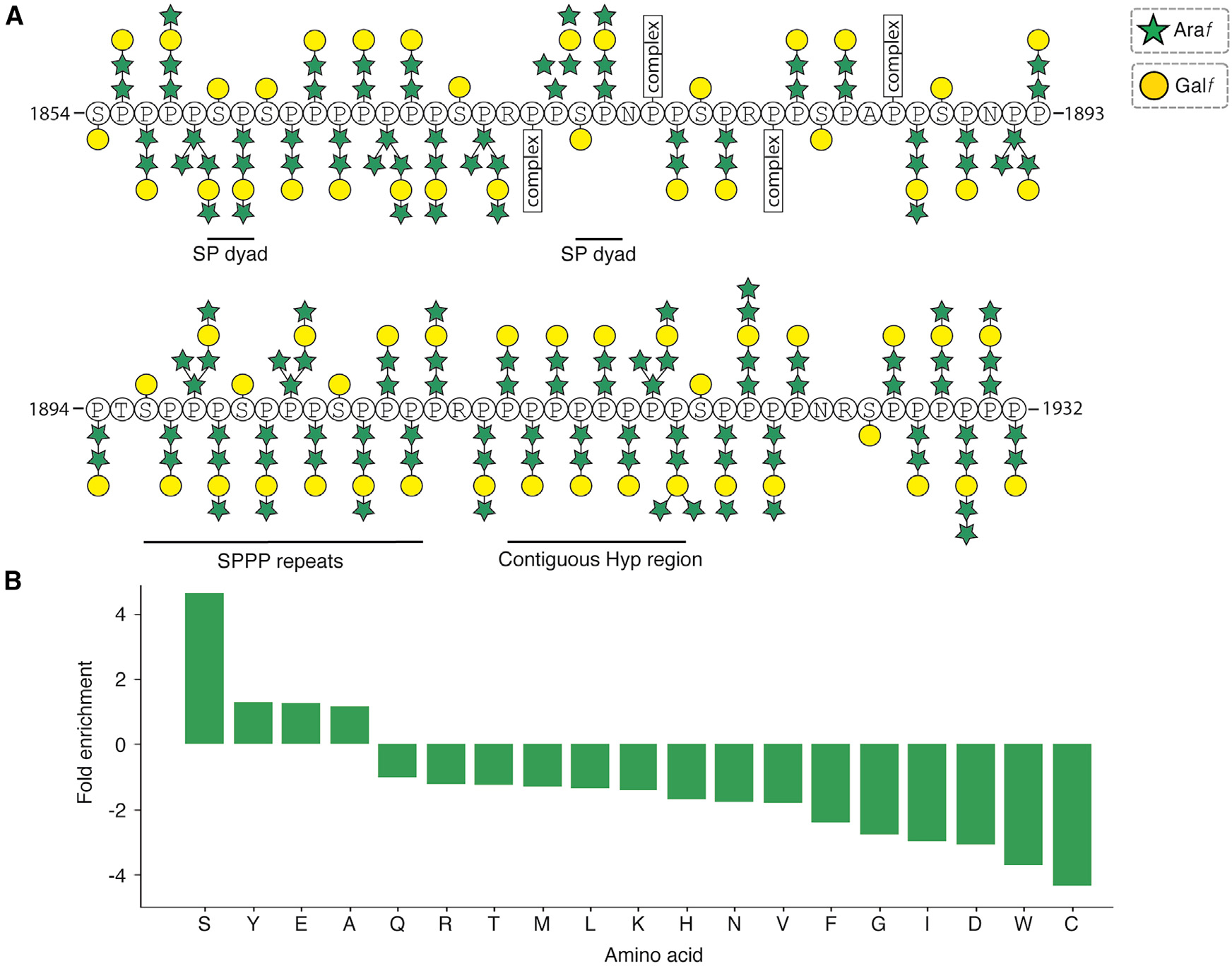
Glycosylation patterns observed for the MST1 PPII helix (A) Schematic showing the glycan structures associated with each hydroxyproline and serine residue of the poly(proline)-rich region of MST1 (residues 1,854–1,932). Saccharides are represented following the Symbol Nomenclature for Glycans (SNFG). Note that these assignments are only tentative, as the cryo-EM density does not allow individual sugar types to be distinguished. The number of saccharide units represents either the exact number or the minimum that can be confidently identified from the cryo-EM density. Large branched glycans are labeled as “complex.” Non-proline/serine residues are not modified. (B) Histogram showing the fold enrichment of each non-proline amino acid in poly(proline)-rich regions of the *C. reinhardtii* proteome. Poly(proline)-rich regions were defined as 20-residue nonoverlapping regions containing 10 or more proline residues. Only serine (S), tyrosine (Y), glutamate (E), and alanine (A) are enriched in poly(proline)-rich regions compared with their general frequency in the proteome.

The density for each glycan is relatively robust, suggesting a high degree of homogeneity. We therefore hypothesize that each glycosylated residue is consistently decorated with glycans that have the same sugar sequence, linkage type, and anomeric configuration. Analysis of the patterns of glycosylation and the underlying amino acid sequence (Figure 4A) indicates that contiguous hydroxyproline residues are more likely to be uniformly decorated with linear trisaccharides rather than more complex glycans. Conversely, hydroxyprolines that are within two residues of a neighboring non-proline are susceptible to more complex glycosylation.

### PPII-localized serine residues are O-galactosylated

Interspersed within the hydroxyproline residues of MST1’s PPII helix are 13 serine residues. The density around the sidechain of each is larger than can be explained by a single hydroxyl group (Figure 3E). The most likely interpretation is that each serine is *O*-glycosylated with a single galactose moiety (Figure 3F), which is a plant- and algal-specific post-translational modification found on members of the HRGP family.^40^ Consistent with this assignment, monogalactosyl serine (Ser-*O*-αGal) has also been observed in crude cell wall preparations from *C. reinhardtii*, and the enzyme responsible, peptidyl serine *O*-α-galactosyltransferase, has been identified.^41^ We have tentatively built the monogalactosyl moiety in its pyranose conformation, which positions the hydroxyl group on carbon 6 such that it can hydrogen bond with the carbonyl group of the backbone of the PPII helix (Figure 3E).

The presence of Ser-*O*-αGal in the poly(hydroxyproline) region is consistent with prior observations that flanking proline residues enhances *O*-linked glycosylation of serine residues.^42^ The fact that every serine residue within the PPII helix is galactosylated suggests that conformation rather than sequence determines whether they are modified.

Four arginine and three asparagine residues are also present within MST1’s PPII helix. We therefore considered whether they, like serine residues, might be overrepresented in proline-rich regions. However, an analysis of the *C. reinhardtii* proteome revealed that only three other residues besides serine (tyrosine, glutamate, and alanine) are enriched (Figure 4B). Interestingly, one of the asparagine residues occurs in a consensus sequence (N^1924^RS) strongly predicted to be *N*-glycosylated, but the density clearly shows that this residue is unmodified (Figure S3E). The formation of a PPII helix may therefore favor *O*-linked over *N*-linked glycosylation. Other residues outside the PPII helix with similar NxS/T consensus sites are *N*-glycosylated: one in D6 (N851) and two in the cysteine-rich domain (N1194 and N1643) (Figures S3A–S3D). Of these, the branched glycan attached to N1643 is the best resolved and the only site that is conserved across MST1-like proteins of the *Chlorophyceae* family. On the basis of prior work, we have modeled the first two moieties of each glycan as *N*-acetylglucosamine (GlcNAc) polymerized through β(1,4)-linkages (Figures S3B–S3D) but left the rest unmodeled due to the compositional complexity of *N*-glycans in *C. reinhardtii*.^43^

### The mastigoneme contains a second poly(hydroxyproline) protein

**Figure 5.**
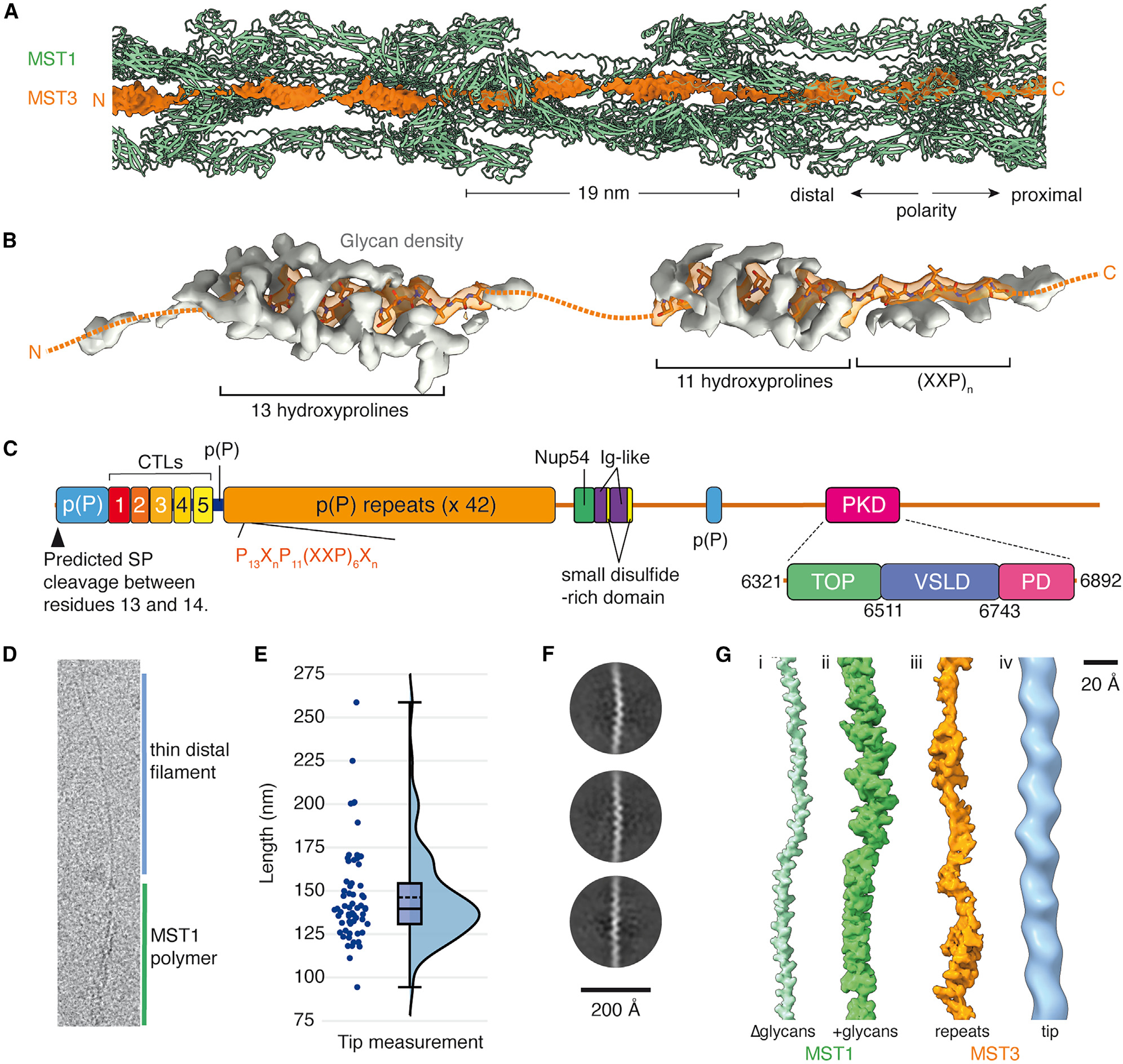
Proline-rich repeats of MST3 occupy the central shaft of the mastigoneme (A) Cryo-EM density (orange) that cannot be explained by MST1 (green model) occupies the central shaft of the mastigoneme. The density does not follow D1 symmetry and repeats approximately every 19 nm. (B) Density map of the 19-nm repeat of the glycosylated poly(hydroxyproline) (PPII) helix of MST3. The density map is segmented to show the protein residues(orange) and the decorating glycans (gray). Glycans bound to XXP motifs coat only one surface of the helix. (C) Schematic of the domain architecture of MST3. Domain boundaries were determined from AlphaFold2 predictions. Abbreviations: CTL, C-type lectin; Ig-like, immunoglobulin-like; PD, pore domain; PKD, polycystic kidney disease; p(P), poly(proline)-rich; VSLD, voltage-sensor-like domain. (D) An example of a mastigoneme with a thin distal filament extracted from a micrograph. (E) Raincloud plot showing the distribution of tip filament lengths measured from the micrographs. Each point represents an individual measurement (n = 62 from50 micrographs). In the box plot, the central dashed line represents the median and the solid line the mean. The bottom and top of the box represent the first (Q1) and third (Q3) quartiles, respectively. The whiskers extend to the highest and lowest scores. (F) Two-dimensional class averages of particles selected from the mastigoneme thin-end filaments. (G) Comparison of a three-dimensional reconstruction of the mastigoneme end filament (iv) with the PPII helices of the central poly(proline) repeats of MST3 (iii) and MST1 with (ii) and without (i) glycans. The overall dimensions of the end filament are consistent with a glycosylated PPII helix. See Figure S4.

Contrary to earlier expectations,^11,44^ we observed that MST1 is not the only protein present in mastigoneme filaments. Our analysis revealed unassigned density occupying the central shaft that strongly resembles, and can be partially modeled by, a glycosylated PPII helix (Figure 5A). This coaxial PPII helix had weaker density than the neighboring PPII helix of MST1, leading us to speculate that it might not follow the same symmetry as MST1. Consistent with this hypothesis, reverting to C1 symmetry during processing (Figure S1B and STAR Methods) improved the local map quality revealing two segments of well-resolved density connected by less well-resolved density (Figure 5B). The first segment corresponded to 13 glycosylated hydroxyproline residues, while the second corresponded to approximately 11 glycosylated hydroxyproline residues followed by a XXPXXPXXP motif, where X is any non-proline residue. A search of the *C. reinhardtii* proteome for proteins with P_13_Y_n_P_11_(XXP)_3_ motifs identified Cre06.g309951 as containing 42 copies (Figure 5C). These repeating motifs are connected by ~20 additional residues of relatively divergent but proline-rich sequence. Mass spectrometry confirmed the presence of Cre06.g309951 in samples of purified mastigonemes (Tables S2 and S3; Figure S4). We therefore rename Cre06.g309951 as MST3.

We next speculated that MST3, a protein with 8,572 residues, might span the entire length of the mastigoneme, with each of the 42 repeats surrounded by MST1. Averaging of these repeats during processing would explain why the density for the conserved P_13_ and P_11_(XXP)_3_ motifs was stronger than the more divergent connecting elements. Furthermore, as each repeat spans a distance of 19 nm (calculated from the cryo-EM density), 42 copies would give a total length of ~800 nm, which is approximately the measured length of mastigonemes.^11,15,44^ The poly (proline) repeats of MST3 might therefore function to set the maximum length of the mastigoneme.

In addition to the 42 poly(proline) repeats, MST3 has a 429-residue poly(proline)-rich N terminus following a likely cleaved signal peptide (Figure 5C). The density of prolines within this region is exceptionally high, such that proline sequences are never punctuated by more than one non-proline residue. Only four of these non-proline residues are not serine, glutamate, or alanine, consistent with our analysis of the whole proteome (Figure 4B). We hypothesized that this region would also form a glycosylated PPII helix but would be unable to bind MST1 due to its different pattern of proline repetition. We further speculated that this region might explain the thinner filament observed at the distal end of mastigonemes.^16,17^ To test this, we manually selected only the fine end filaments from our micrographs (an example of which is provided in Figure 5D). The mean length of the filaments was 146 nm (Figure 5E), which is close to the theoretical distance (133 nm) that can be generated by 429 residues adopting a continuous PPII helix. Longer filaments may correspond to MST3 exposed by depolymerization of MST1. Two-dimensional classification of particles extracted every ~110 Å along the filaments revealed projections consistent with a single glycosylated PPII helix (Figure 5F), further supported by comparing a three-dimensional reconstruction with the glycosylated PPII helices of MST1 and MST3 (Figure 5G).

This information establishes that the N terminus of MST3 is at the distal end of the mastigoneme and that the mastigoneme has polarity even if MST1 does not. It also demonstrates that PPII helices are sufficiently stable to form filaments capable of spanning hundreds of nanometers.

### MST3 contains a PKD-like domain

**Figure 6.**
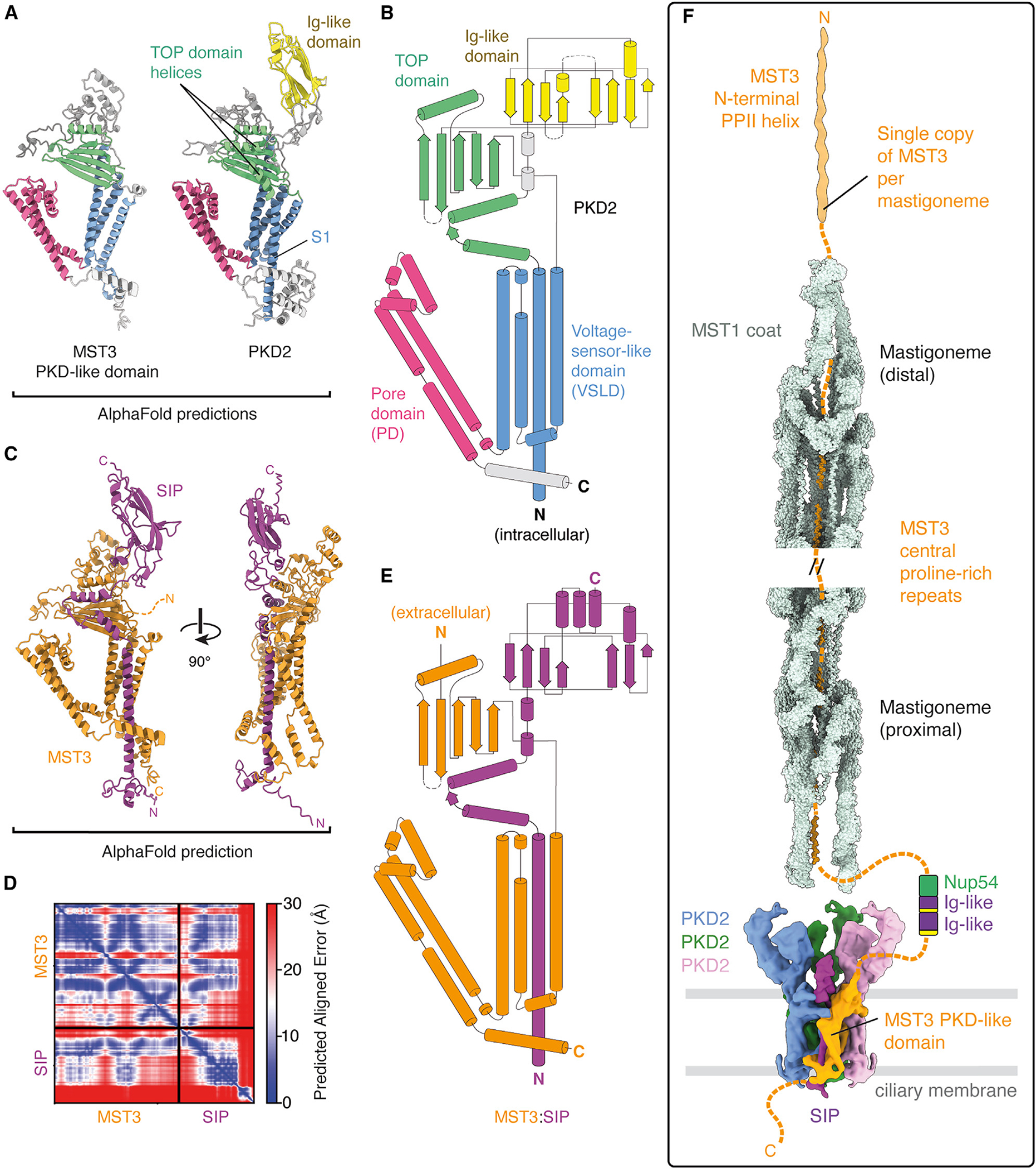
*C. reinhardtii* MST3 contains a PKD-like domain (A) Comparison of AlphaFold2 predictions of the PKD-like domain of MST3 with PKD2. Compared with PKD2, MST3 is missing helix S1 of the transmembrane voltage-sensor-like domain (blue), two helices of the juxtamembrane TOP domain (green), and the extracellular immunoglobulin (Ig)-like domain (yellow). The pore domain (pink) is highly similar. (B) Topology diagram of *C. reinhardtii* PKD2 colored by domain and generated from the AlphaFold2 prediction. Cylinders represent α-helices, and arrows represent β-strands. (C) AlphaFold2 prediction of the complex between the PKD-like domain of MST3 and SIP. (D) Predicted aligned error (PAE) plot for the prediction shown in (C). High-confidence interactions are predicted between MST3 and SIP. (E) A topology diagram generated from the AlphaFold Multimer prediction of the MST3:SIP complex colored by protein. (F) Integrated model of the mastigoneme combining our cryo-EM data with AlphaFold2 predictions. One copy of the MST3:SIP complex (orange and purple) anchors the mastigoneme in the ciliary membrane through interactions with 3 identical copies of PKD2 (shown in blue, green, and pink). The MST1 coat around the MST3 proline-repeats likely extends 800 nm and includes ~84 copies of MST1 (based on 4 copies per 380 Å). The N terminus of MST3 forms a 140-nm PPII helix at the distal end of the mastigoneme. Its 1,600-residue intracellular C terminus may be responsible for anchoring the mastigoneme to specific doublet microtubules of the axoneme.

Bioinformatic analysis revealed that MST3 contains a transmembrane PKD-like domain (Figure 5C) that is structurally and topologically similar to *C. reinhardtii* PKD2 (Figures 6A and 6B). As PKD channels are typically homo- or heterotetramers,^45^ a subunit formed by MST3 could potentially explain how mastigonemes are tethered to PKD2-containing complexes in the ciliary membrane.^16^ However, closer inspection revealed that the PKD domain of MST3 lacks transmembrane helix 1 (S1) of the voltage-sensor-like domain, two helices that pack against the 5-stranded β-sheet of the juxtamembrane TOP domain (also known as the polycystin domain^46^), and an extracellular Ig-like domain (Figure 6A). Remarkably, this exact missing region is encoded by SIP, a small protein recently identified to copurify with *C. reinhardtii* PKD2.^19^ We therefore speculated that SIP could bind MST3 to complete the PKD fold. Consistent with this idea, an AlphaFold2 multimer prediction of the MST3:SIP complex bears a striking resemblance to PKD2 (Figures 6C–6E). A role for SIP in anchoring mastigonemes to PKD2 in the membrane explains the lack of mastigonemes in *sip* mutants.^19^ Taken together, we propose that PKD2, MST3, and SIP form a complex in the ciliary membrane with a 3:1 stoichiometry between PKD2 and the MST3:SIP complex.

To test the interdependence of MST1, MST3, SIP, and PKD2 for mastigoneme formation, we obtained and validated (Figures S4D–S4F) mutants of each from the Chlamydomonas Library Project (CLiP).^47^ Using negative-stain electron microscopy, we first confirmed that the *mst1, sip*, and *pkd2* mutants lack mastigonemes (Figure S5A), as previously reported.^16,19^ We next examined four different *mst3* mutants and observed that all lack mastigonemes (Figures S5B and S5C), consistent with the proposed central role of MST3 in mastigoneme formation. The *mst3* mutant strains also displayed a modest (~15%) but statistically significant reduction in swimming velocity compared with wild-type (WT) strain (Figure S6A) that is similar to the reduction reported in some studies with *mst1, pkd2*, and *sip* mutants.^16,19^

**Figure 7.**
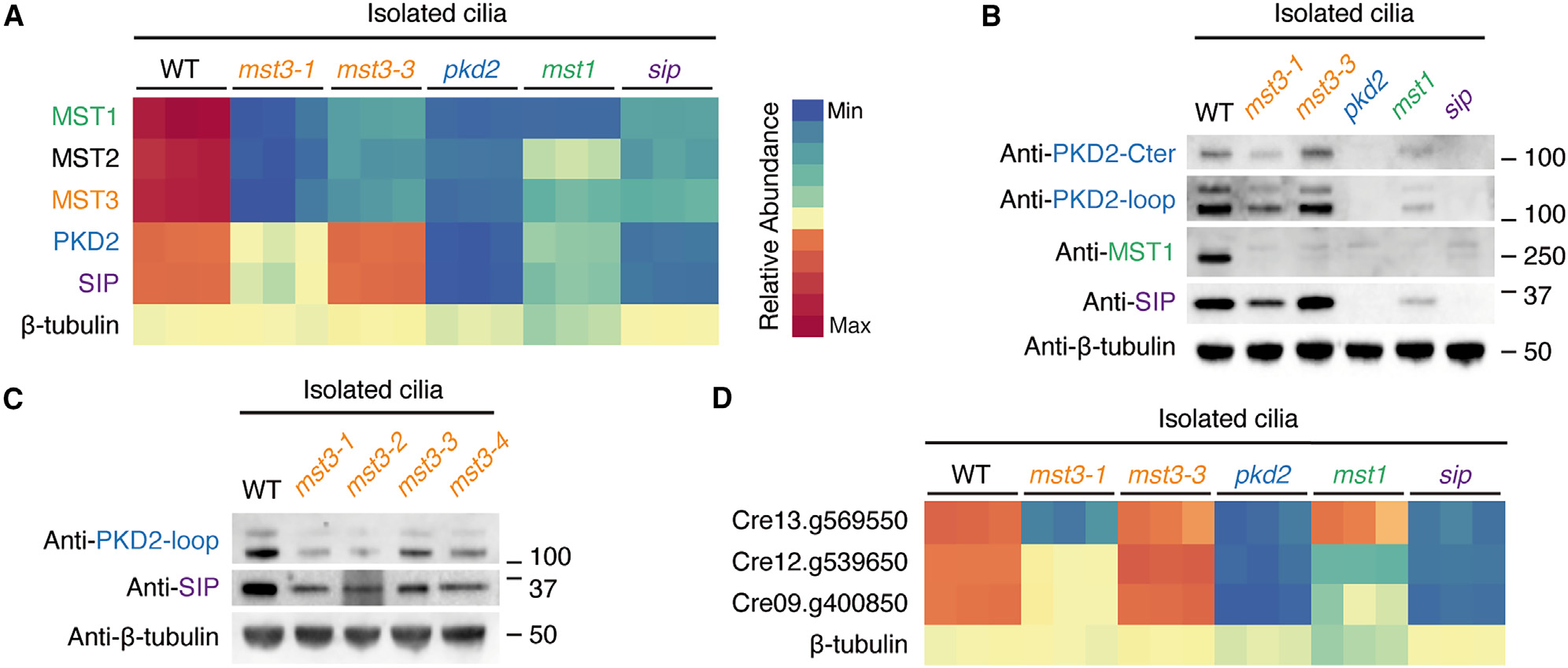
Proteomic comparison of cilia composition from *C. reinhardtii* mutant strains (A) Heatmap comparing the relative abundance of MST1, MST2, MST3, PKD2, and SIP in proteomic analysis of cilia isolated from wild-type (CC-5325) and mutant (*mst3–1, mst3–3, pkd2, mst1*, and *sip*) strains. The relative abundance of b-tubulin is provided as a control. (B) Immunoblot of cilia isolated from WT, *mst3–1, mst3–3, pkd2, mst1*, and *sip* strains probed with antibodies against PKD2-Cter, PKD2-loop, MST1, and SIP. Anti-β-tubulin was used as a loading control. Anti-PKD2-Cter detects two bands due to PKD2 proteolysis, as previously reported.^19,48^ (C) Immunoblot of cilia isolated from WT, *mst3–1, mst3–2, mst-3*, and *mst3–4* strains probed with antibodies against PKD2-loop, SIP, and β-tubulin. PKD2 and SIP levels are reduced to varying extents in each *mst3* strain. (D) Heatmap generated from quantitative mass spectrometry data showing that the relative abundance of three predicted SIP binders (Cre13.g569550,Cre12.g539650, and Cre09.g4008500) is reduced in *pkd2* and *sip* strains. Four other predicted SIP binders (listed in Figure S7) were not detected. See Figures S4, S6, and S7.

We next performed multiplex quantitative mass spectrometry and immunoblotting of cilia isolated from the parental strain used for the CLiP collection (CC-5325), two of the *mst3* strains (*mst3–1* and *mst3–3*), as well as from the *mst1, sip,* and *pkd2* mutants (see STAR Methods). These data (Figures 7A, 7B, and S6B) confirmed the absence of MST1 in all strains except WT, consistent with the visible loss of mastigonemes from ciliary membranes (Figure S5). Interestingly, the MST1 paralog, MST2, was reduced in all five mutants, suggesting that at least some mastigonemes contain MST2. MST3 levels were also severely reduced in the *pkd2* and *sip* mutants, consistent with our proposed model that MST3 binds SIP and that this complex interacts with PKD2. MST3 was also reduced in the *mst1* strain, suggesting that MST3 is unstable without MST1. Consistent with this dependence on MST1, we found no evidence of thin exterior filaments that might correspond to undecorated MST3 during negative-stain electron microscopy analysis of *mst1* mutant cilia (Figure S5A).

We also observed that *mst3–1* and *mst3–3* differ in their levels of SIP and PKD2. *mst3–1* has reduced levels of both proteins, while *mst3–3* have levels of SIP and PKD2 similar to WT. Given the variation between strains, we used immunoblot analysis to examine the two other MST3-deficient strains (*mst3–2* and *mst3–4*) (Figure 7C). The results showed that, in general, SIP and PKD2 levels are reduced in *mst3* strains but not completely absent, demonstrating that SIP and PKD2 are not fully dependent on MST3 for their entry into cilia.

## DISCUSSION

Our structure of the *C. reinhardtii* mastigoneme filament demonstrates that it consists of multiple copies of MST1 around a central single copy of MST3. A different cryo-EM study of the same complex,^44^ published while our paper was under consideration, only identified MST1. However, unassigned density corresponding to MST3 is clearly present in their deposited map (EMDB: EMD-41679). The identification of a single copy of MST3 in each mastigoneme establishes that mastigonemes have polarity and provides explanations for how mastigoneme length is determined, why mastigonemes have narrow distal tips, and how mastigonemes are anchored to PKD channels in the ciliary membrane.

Within the membrane, our bioinformatic analysis supports a model where the PKD-like domain of MST3 binds SIP, and together they form a complex with PKD2 with pseudo tetrameric symmetry (Figure 6F). A 3:1 stoichiometry between PKD2 and MST3:SIP would explain why each PKD channel is only associated with a single mastigoneme.^16^ It would also match the stoichiometry observed for the human PKD1-PKD2 channel, ^49,50^ mutations in which are a major cause of autosomal dominant polycystic kidney disease. Indeed, MST3 (and the mastigoneme in general) may function analogously to human PKD1, as both have large N-terminal extracellular regions that contain multiple Ig-like domains and one or more putative carbohydrate-binding C-type lectin (CTL) domain. ^51^

We propose that MST3 and SIP arose through fragmentation of a progenitor PKD2-like gene. During this fragmentation event, the first helix of the PKD domain became encoded by a separate gene (*SIP*), which changed the orientation of the N terminus of the remaining transmembrane domain from intracellular to extracellular. The extracellular orientation allowed the N terminus of the PKD transmembrane domain to acquire additional domains, presumably through gene fusions. In addition to MST3, we identified 7 other proteins in *C. reinhardtii* with large extracellular regions and incomplete PKD domains that can be completed with SIP based on AlphaFold Multimer predictions (Figures S7A and S7B). Three of these proteins (Cre13.g569550, Cre12.g539650, and Cre09.g400850) are expressed in cilia and have significantly reduced abundancies in *sip* and *pkd2* mutant strains (Figure 7D), supporting their annotation as SIP-interacting proteins. Whether splitting transmembrane folds to alter terminal orientations is a common evolutionary mechanism in other species remains to be investigated. Alternatively, acquiring an odd number of additional transmembrane helices could achieve the same reorientation, as seen with the elaborate extracellular N terminus of mammalian PKD1, which contains five additional transmembrane helices beyond the PKD domain. ^49^ The prevalence of carbohydrate-binding domains within this cohort of proteins, along with MST3 and human PKD1, suggests a general role for carbohydrate binding in the function of PKD channels.

### Insights into the hydroxyproline glycosylation code

The structure of the native *C. reinhardtii* mastigoneme allows visualization of the glycan patterns associated with different types of proline repetition in two different HRGPs (MST1 and MST3), providing insight into the hydroxyproline glycosylation code.^9^ From our structure, we propose six general rules:

1. Proline residues within PPII helices are converted to *O*-linked glycosylated hydroxyprolines regardless of sequence context. We note that there may be exceptions to this rule, as prior work has suggested that lysine—an amino acid that does not occur in the PPII helix of MST1 or the MST3 N terminus—can inhibit proline hydroxylation,^6^ and 5% of cellular hydroxyproline in crude extracts of *C. reinhardtii* cell walls was not glycosylated.^32^ However, all the proline residues in the mastigoneme structure appear to be fully glycosylated.
2. Serine residues interspersed among hydroxyproline residues are monogalactosylated regardless of their flanking sequences or motif they occur in (Figure 4A). *O*-linked galactosylation of serine residues is therefore dependent on peptide configuration rather than specific sequence motifs. The function of monogalactosylated serine within PPII helices is likely to rigidify the protein backbone, which is characteristically devoid of internal hydrogen bonding, by hydrogen bonding with a preceding peptide bond (Figure 3E).
3. Hydroxyproline residues within contiguous proline stretches are more likely to be uniformly glycosylated than those within two residues of a neighboring non-proline residue, which are more likely to have complex, branched glycans (Figure 4A). The inclusion of non-proline residues (including, to a lesser extent, serine) may therefore introduce opportunities to generate more diverse glycans than stretches of contiguous prolines (Figure 4A).
4. High proline density in PPII helices (i.e., an average of 2 or 3 prolines per every 3 residues) allows glycans to wrap around the PPII helix to encase the helix backbone in carbohydrate. The ability to form this carbohydrate casing is perhaps more important than the underlying proline sequence, as the PPII helix of MST2 has 22 single-residue substitutions compared with MST1 but only 4 fewer prolines (Figure S2L).
5. Repetitive XXP motifs (as found in MST3) create PPII helices with two distinct faces: one face dominated by glycosylated hydroxyproline and the other by protein sidechains (Figure 5B). PPII helices formed from XXP motifs therefore have very different properties and can form different interactions than those formed from contiguous prolines and proline residues in XP dyads and XPPP(P) motifs.
6. Non-proline residues within PPII helices introduce flexibility (in addition to glycan complexity) and divide the helix into discrete “modules.” This is based on the observation that glycosylated regions of the MST1 PPII helix are straighter than those that feature one or more non-glycosylated residues (Figure 3A).

### Functional implications of glycosylated PPII helices

Overall, the function of *O*-linked glycans in PPII helices is likely to rigidify the protein backbone, which are characteristically devoid of internal hydrogen bonding. A stabilizing function for the carbohydrate is consistent with circular dichroism spectroscopy experiments showing that deglycosylation destabilizes the PPII conformation. ^52^ The additional stability provided by glycans is likely sufficient to allow PPII helices to exist extracellularly as extended rod-like structures, as appears to be the case for the N-terminal helix of MST3 (Figure 5). Here, the glycosylation likely not only rigidifies the helix but also potentially protects it from enzymatic digestion by aminopeptidases. This may be a general protective mechanism, as there are 100 other proteins in the *C. reinhardtii* proteome that have N-terminal poly(proline)-rich regions (25 or more prolines within the first 100 residues).

The structure of the mastigoneme also demonstrates that repetitive poly(proline) regions can function as molecular rulers, providing a permanent scaffold that other proteins can bind to. For example, the repetitive poly(proline) regions of MST3 bind MST1 and set the maximum length of the filament, ensuring a relatively uniform distribution of lengths among mastigoneme filaments. Measuring and maintaining distance between adjacent repeat units may also explain the role of the PPII helix in MST1 filamentation, where it connects adjacent repeat units (Figures 2D and 2E).

Based on sequence differences between the N terminus of MST3 (which forms a free PPII helix) and the PPII helices of MST1 and the MST3 poly(proline) repeats, we propose that regions of high proline density, especially those that lack non-proline insertions, are likely to form exposed rod-like PPII helices, while those with more intricate patterns of proline repetition are more likely to bind partner proteins.

### Limitations

Our structure of the mastigoneme has revealed the native glycosylations that can occur on the serine and hydroxyproline residues of PPII helices. Although the quality of our density allows discrimination between linear and branched chains and estimations of the number of saccharide moieties in each glycan, mass spectrometry or cryo-EM resolutions closer to 2 Å will be needed to sequence each glycan precisely and identify enantiomers, ring structures, and linkage types. Both approaches are technically challenging. More structures of other HRGPs will be necessary to fully decipher the rules of the hydroxyproline glycosylation code, which may have organism- or tissue-specific variations. Our work demonstrates the role that cryo-EM is likely to have in this process. Although we identified a link between mastigonemes and the PKD channel, further experiments, including crosslinking mass spectrometry and structural biology, will be necessary to validate our model. Understanding this interaction may help to uncover a function for the mastigoneme and even possibly the human PKD1-PKD2 channel, with which it shares common features. How mastigonemes are tethered to specific doublet microtubules of the axoneme, and if the long intracellular C terminus of MST3 plays a role, also remains a mystery.

## STAR★METHODS

### RESOURCE AVAILABILITY

#### Lead contact

Further information and requests for resources should be directed to and will be fulfilled by the lead contact, Dr. Alan Brown (alan_brown@hms.harvard.edu)

#### Materials availability

Wild-type *Chlamydomonas reinhardtii* cells (CC-1690, CC-4402, and CC-5325) and sip (LMJ.RY0402.143879), *pkd2* (LMJ.RY0402.204581), *mst1* (LMJ.RY0402.052413) and *mst3* (LMJ.RY0402.120927, LMJ.RY0402.226217, LMJ.RY0402.079427, and LMJ.RY0402.104433) mutants are available from the Chlamydomonas Resource Center (University of Minnesota).

#### Data and code availability

- A composite cryo-EM map of the *C. reinhardtii* mastigoneme has been deposited in the Electron Microscopy Data Bank (EMDB) with accession code EMD-43892. Constituent maps have been deposited with accession codes EMD-43889, EMD-43890, and EMD-43891. An atomic model of the mastigoneme has been deposited in the Protein Data Bank (PDB) with accession code 9B4H. Mass spectrometry data of purified mastigonemes are available as Table S2. Images used to calculate the swimming velocity of *C. reinhardtii mst3* mutants relative to wild type is available at https://zenodo.org/records/10627574. Quantitative mass spectrometry data comparing mutant strains has been deposited to PRIDE^74^ with accession code PXD049250.
- Bespoke code used to identify MST3 and the residues enriched in poly(proline)-rich regions are available at https://zenodo.org/records/10093208.
- Any additional information required to reanalyze the data reported in this paper is available from the lead contact upon request.

### EXPERIMENTAL MODELS AND STUDY PARTICIPANT DETAILS

Wild-type *C. reinhardtii* strains (CC-1690, CC-4402 and CC-5325) were obtained from the Chlamydomonas Resource Center. CC-1690 cells were used to purify mastigonemes. CC-4402 cells were used to prepare cryo-EM samples. CC-5325 is the background strain used in the Chlamydomonas Library Project (CLiP) ^47,55^ and was used for comparative studies with mutants. Four *mst3* mutants were analyzed: *mst3–1* (LMJ.RY0402.079427), *mst3–2* (LMJ.RY0402.104433), *mst3–3* (LMJ.RY0402.120927), and *mst3–4* (LMJ.RY0402.226217). Mutants for *sip* (LMJ.RY0402.143879), *pkd2* (LMJ.RY0402.204581) and *mst1* (LMJ.RY0402.052413) were also obtained from the CLiP collection and had been characterized previously.^19^ All strains were cultured in TAP (Tris-acetic acid-phosphate) or M media.

### METHOD DETAILS

#### Genetic characterization of CLiP mutants

*C. reinhardtii* genomic DNA was extracted from wild-type and *mst3* mutant strains using QuickExtract DNA extraction solution (Biosearch Technologies). Cells grown on TAP plates were suspended in the solution and incubated at 65°C for 6 min and 98°C for 2 min. PCR reactions were performed using Q5 (BioLabs) and DreamTaq (Thermo Scientific) DNA polymerases following the manufacturer’s protocols. Primers are listed in Table S4. Annealing temperatures (between 55 and 71°C) were determined using the NEB Tm calculator (v.1.16.5). PCR cycle number was set to 31.

#### Cilia isolation

Cilia were isolated from *C. reinhardtii* strains using a modified version of the dibucaine method. ^75^ Briefly, cells grown in TAP or M media were harvested and washed at least twice with 10 mM HEPES pH 7.4. Cilia were detached by resuspending the cells in ice-cold HMS buffer (10 mM HEPES, 5 mM MgSO_4_, 4% sucrose) with 4.2 mM dibucaine and pipetting. The treatment was quenched by adding HMS buffer containing 0.5 mM EGTA. Cell bodies were removed by centrifugation at 1200 × g for 3 min, and further separated by underlaying the supernatant with a 25% sucrose cushion and centrifugation at 1700 × g for 10 min. Isolated cilia were then sedimented by centrifugation at 12,000 × g for 15 min for mastigoneme isolation, quantitative proteomics, and immunoblot analysis.

#### Mastigoneme isolation

Mastigonemes were isolated from cilia from wild-type CC-1690 cells using a modified centrifugation-based method.^11^ Cilia pellets were resuspended in 10 mM Tris-HCl pH 7.8 with 0.7% Sarkosyl and protease inhibitor and incubated at 4°C for 6–8 h. The sample was centrifuged at 111,000 × g (SW 41 Ti rotor) for 45 min. The pellet was resuspended in 2.8 M CsCl in 10 mM Tris-HCl pH 7.8 and centrifuged at 134,000 × g (SW 41 Ti rotor) for 20 h. Mastigonemes appeared near the center of the gradient, forming a narrow white band. Fractions were taken and examined using negative stain electron microscopy for the presence of mastigonemes. The target fraction was diluted 1:1 with 10 mM Tris-HCl pH 7.8 and spun down briefly to remove debris. The supernatant was then centrifuged at 111,000 × g (SW 41 Ti rotor) for 45 min to pellet purified mastigonemes.

#### Negative-stain electron microscopy

Isolated mastigonemes (0.2–0.7 mg/mL) or whole *C. reinhardtii* cells (10^7^ cells/mL) were applied to glow-discharged carbon-coated copper grids (Electron Microscopy Sciences). After 1 min incubation, the grids were blotted, immediately washed twice with 1.5% uranyl formate solution, and stained for 90 s with 1.5% uranyl formate solution. The grids were examined using a 120 kV Tecnai T12 (Thermo Fisher Scientific) microscope. Images were recorded using an Ultrascan 895 CCD camera (Gatan).

#### Mass spectrometry analysis of mastigonemes

Isolated mastigonemes were denatured by adding sodium dodecyl sulfate(SDS)-loading buffer. The denatured samples were then loaded onto a 4–20% SurePAGE Bis-Tris gel (GenScript) and electrophoresed for 40 to 70 min. The gel was stained with SyproRuby (Sigma-Aldrich) before excising bands for in-gel trypsin digestion. Gel pieces were washed and dehydrated with acetonitrile for 10 min. After completely drying in a speed vacuum, the gel pieces were rehydrated with 50 mM ammonium bicarbonate solution containing 12.5 ng/μL of sequencing-grade trypsin (Promega). After 45 min at 4°C, the trypsin solution was removed and replaced with 50 mM ammonium bicarbonate solution to just cover the gel pieces. Samples were incubated at 37°C overnight. Peptides were later extracted by removing the ammonium bicarbonate solution, followed by one wash with a solution containing 50% acetonitrile and 1% formic acid. The extracts were then dried in a speed vacuum for ~1 hr and stored at 4°C until analysis.

On the day of mass spectrometry analysis, dried peptide samples were reconstituted in 5–10 μL of solvent A (2.5% acetonitrile, 0.1% formic acid). The peptides were then loaded onto a pre-equilibrated nano-scale reverse-phase HPLC capillary column (100 μm inner diameter × ~30 cm length) containing 2.6 μm C18 spherical silica beads. A gradient of increasing concentrations of solvent B (97.5% acetonitrile, 0.1% formic acid) was used to elute the peptides using a Famos auto sampler (LC Packings). As peptides eluted from the column, they were subjected to electrospray ionization into a Velos Orbitrap Pro ion-trap mass spectrometer (Thermo Fisher Scientific). Tandem mass spectra were acquired and analyzed using Sequest (Thermo Fisher Scientific) against a protein database containing normal and reversed versions of all sequences to determine peptide identities. Data were filtered to a peptide false discovery rate (FDR) of 1–2%.

#### Tandem mass tag (TMT) labeling and on-bead digestion

Proteins in isolated cilia (3 replicates per *C. reinhardtii* strain) were reduced by the addition of Tris(2-carboxyethyl)phosphine hydrochloride (TCEP) to a final concentration of 10 mM. 20 μL of 500 mM ammonium bicarbonate was added to 100 μL of 3 μg/mL isolated cilia to bring the pH above 8. Cysteine residues were alkylated by adding 5.5 μL of 400 mM iodoacetamide in 50 mM ammonium bicarbonate then incubated for 25 min at room temperature in the dark. The reactions were quenched with 5.5 μL of 1 M dithiothreitol. Protein was extracted using the single-pot, solid-phase-enhanced sample-preparation (SP3) method. ^76^ Briefly, hydrophobic and hydrophilic magnetic beads (Cytiva) were combined in a 1:1 mixture and washed twice with high-performance liquid chromatography (HPLC)-grade water and then resuspended so the final concentration of beads was 50 mg/mL. 60 μL of bead stock was added to each sample. The samples were mixed with gentle pipetting and binding was induced by the addition of 181 μL 200 proof ethanol to achieve a 50% ethanol mixture. The samples were incubated in a thermomixer at 24°C for 5 min at 1000 rpm. The supernatant was removed using a magnetic rack and the beads were washed three times with 80% ethanol.

The proteins were enzymatically digested on the beads with 2 μL of 2 mg/mL Endoproteinase Lys-C (Wako) in 50 μL 200 mM EPPS pH 8.5 with 4% acetonitrile for 3 h while shaking at 37°C. The samples were further digested by adding 1 μL of trypsin (Promega) in 200 mM EPPS pH 8.5 and shaking overnight at 37°C. To ensure that a minimum of 90% tryptic sites were digested, 2 μL of digested protein from each sample were desalted by STAGE tip^77^ and analyzed by LC-MS/MS. Once complete digestion was confirmed, 100 μg of each sample was diluted with 200 mM EPPS pH 8.5 with 2% acetonitrile to achieve 100 μL and further diluted with 30 μL acetonitrile. Samples were labeled with TMTpro isobaric reagents (Thermo Scientific) for 1 hour at room temperature with frequent vortexing. To measure labeling efficiency, 2 μL of each sample was pooled and analyzed by synchronous precursor selection (SPS)-MS3. ^78^ Once labeling was confirmed to be above 95%, samples were quenched with 7 μL 10% hydroxylamine, acidified with formic acid, pooled while adjusting for TMT ratios to be within 80% relative intensity, and dried by speed vacuum to near completion.

The multiplex was resuspended in 0.3 mL 1% formic acid in HPLC-grade water and fractionated using a high pH reversed-phase benchtop fractionation kit (Thermo Scientific) using the following step gradient of increasing percentages of acetonitrile: 10, 12.5, 15, 17.5, 20, 25, 30, 35, 40, 50, 65, and 80%. The twelve fractions were combined into six as follows: 1+7, 2+8, 3+9, 4+10, 5+11, 6+12, dried, desalted by STAGE tip, and resuspended in 1% formic acid with 3% acetonitrile for analysis.

#### Quantitative proteomics acquisition and data analysis

The TMT-labeled peptides were analyzed using an Orbitrap Eclipse Tribrid MS with a FAIMS device and connected to a Vanquish NEO UHPLC (Thermo Fisher Scientific). Fractions were first separated on an EASY-Spray PepMap 75 μm C18 column (Thermo Fisher Scientific) with the following gradient: 5–6% B in 5 min, 6–12% B in 100 min, 12–24% B in 74 min, 24–35% B in 36 min, 35–70% B in 18 min, 70–100% B in 5 min at a flow rate of 250 nL/min. Data were collected using SPS-MS3 with FAIMS (cyclic voltage of −40/−50/−70). The MS1 spectra were collected in the orbitrap (resolution 120,000; a scan range of 400–1500; AGC target of 200, and dynamic exclusion 60 sec). Peptides were fragmented using collision-induced dissociation (CID, CE=35%) and MS2 scans were collected using the ion trap with normalized automatic gain control (AGC) and maximum injection time set to 100% and auto, respectively. Real-time search was used with a *C. reinhardtii* proteome extracted from Phytozome v.6.1 in which the sequence of MST3 was replaced with the hybrid protein sequence (see MST3 identification section below). Static modifications of alkylated cysteine (+57.0215), TMTpro at the N terminus and lysine sidechains (+304.2071) and dynamic modification of oxidized methionine (+15.9949) were permitted. MS3 precursors were fragmented using high-energy collision-induced dissociation (HCD, CE=50%) then analyzed in the orbitrap with a resolution of 50,000. ^79^

Raw spectra were converted to mzXML to perform a post-search calibration. Peptide-spectrum matches (PSMs) were made using Comet (v.2019.01 rev. 5) ^59^ and the GFY Core platform (Harvard University) with the same proteome and modifications used in the real-time search. The database also contained common contaminants and reversed sequences as decoys. The searches were all performed using a mass tolerance of 20 ppm and fragment ion tolerance of 0.9. PSMs were filtered by linear discriminant analysis with a FDR of 1% and a FDR of 1% for collapsed proteins. All MS1 spectra were post-search calibrated and searched again. For TMT quantification, MS2 data were filtered for an isolation specificity above 70% and a minimum summed signal-to-noise of 200 across all TMT channels for each peptide.

#### Immunoblot analysis

Isolated cilia were denatured in LDS-sample buffer (Boston BioProducts) for 10 min at 95C. The denatured samples were then loaded onto a precast 4–20% SurePAGE Bis-Tris gel (GenScript) and separated by electrophoresis in Tris-MOPS-SDS running buffer (GenScript) before being transferred to nitrocellulose membranes (Cytiva). Membranes were blocked with 5% skim milk in TBST (Tris-buffered saline with Tween-20), followed by addition of primary antibodies (Key Resources Table). Antibodies against PKD2-Cterm^48^ and β-tubulin (Cell Signaling Technology, RRID: AB_2210545) were used at a 1:1000 dilution. Antibodies against PKD2-loop, ^48^ MST1,^19^ and SIP^19^ were used at 1:2000 dilution. Primary antibodies were detected with a horseradish peroxidase-conjugated anti-rabbit antibody (Sigma-Aldrich, RRID: AB_92591) at 1:2000 dilution. After washing in TBST, peroxidase substrate (Cytiva) was applied to the membrane and imaged using a Gel Doc XR+ imaging system and the Image Lab software (Bio-Rad).

#### Swimming velocity measurements

*C. reinhardtii* cells were grown to 2 ×10^6^ cell/mL in M medium, washed with fresh M medium, and transferred to chamber slides (Countess, 10 μL/well). The slides were observed using a Nikon Eclipse Ti microscope equipped with a 10 X objective lens. Images were captured at 400 ms exposure time using a CoolSNAP EZ CCD camera and μManager v.1.4 software. ^56^ The swimming paths were analyzed using Fiji. ^65^

#### Cryo-EM data collection

Cryo-EM data used in this study were collected and reported previously.^23^

#### Cryo-EM data processing

Mastigonemes were automatically selected from 19,601 motion-corrected micrographs using ‘Filament Tracer’ in CryoSPARC^24^ with a few templates obtained from mastigonemes manually picked from 50 randomly selected micrographs. The step size between adjacent particle boxes was arbitrarily chosen as 70 Å. Two-dimensional classification was used to remove non-mastigoneme particles, typically doublet microtubules (Figure S1A). Refinement was performed in five steps. In step 1, we used the ‘Ab-Initio Reconstruction’ function in CryoSPARC to generate an initial model and performed ‘Homogeneous Refinement’ in CryoSPARC with no symmetry applied. The resulting 3D reconstruction at 4.3 Å resolution revealed helical symmetry (rise 380 Å, twist 172°). In step 2, we re-extracted the particles using a larger box size (1024 pixels, 1423.4 Å, 2x binning) to accommodate the large helical rise and used ‘Helical Refinement’ function in CryoSPARC to obtain a 3D reconstruction at 6.7 Å resolution. A helical symmetry of (rise 380.16 Å, twist 172.48°) was applied and refined during the helical reconstruction process. The resulting structure revealed an additional D1 point group symmetry, with a symmetry axis perpendicular to the helical axis (Figure 1). In step 3, we used the alignment parameters obtained from step 2 to re-extract the particles using a smaller box size (512 pixels, 711.7 Å, no binning), removed duplicate particles whose centers are too close to each other using ‘Remove Duplicate Particles’ function in CryoSPARC, and performed refinement using ‘Homogeneous Refinement’ in CryoSPARC with D1 symmetry applied, reaching ~3.8 Å resolution. In step 4, we did D1 symmetry expansion (which doubled the particle number) and performed local refinement using all the particles. Then we did local CTF refinement and another round of local refinement, resulting in ~3.4 Å resolution. In step 5, we continued the local refinement (with non-uniform refine enabled^25^) using short cylindrical masks (180 Å in diameter, 200 Å in length) that cover the top, middle and bottom portions of the reconstruction box (Figure S1A), resulting in resolutions of ~3.0–3.1 Å. These local refined maps were aligned to the consensus map from step 2 and stitched together in ChimeraX^60^ using the *vop maximum* command. B-factor map sharpening was performed in cryoSPARC. The final structure came from 687,452 symmetry-expanded particles from two Krios microscopes (Table S1). To allow merging, particles from dataset 2 were rescaled to the pixel size of dataset 1 (1.39 Å) using relion_image_handler.^72^

In the mastigoneme structure, the central stalk density was weaker than the neighboring MST1 density. As one likely cause was that the corresponding protein did not follow the same D1 symmetry as MST1, we subtracted MST1 protein densities from the raw particles images and performed 3D classification of the central stalk density using ‘3D classification’ function in CryoSPARC. The 3D classification resulted in two distinct classes related by D1 symmetry, demonstrating that the central stalk does not follow the same D1 symmetry as MST1 (Figure S1B). In the final step, we merged all the particles from the two D1-symmetry related classes as revealed by 3D classification (by rotating particles from one class to match the 3D orientation of the other class) and computed the final structure with C1 symmetry. As with the symmetry expanded map, focused refinements were performed to generate a composite with improved local resolution. This map was used for model building, refinement, and deposition.

#### Cryo-EM processing (distal tip)

The extended thin filaments at the distal tip of the mastigoneme were manually picked from 1,303 motion-corrected micrographs using ‘Manual picker’ in CryoSPARC. In total 14,758 particles were extracted using a box size of 256 pixels (355.8 Å) and a step size of 80 pixels (111.2 Å). We then performed 2D classification of these particles, which revealed a zig-zag appearance consistent with a poly(proline) helix (Figure 5F). Next, we generated an initial model from the best classes (4,015 particles) using the ‘Ab-Initio Reconstruction’ function in CryoSPARC. Finally, we refined the structure of the distal tip using the ‘Helical Refinement’ function in CryoSPARC with no symmetry applied (Figure 5G).

#### Mastigoneme tip length measurements

The extended tips of mastigonemes were measured using manual tracing in Fiji.^65^ Only tips where the start and end points could be clearly observed were measured. In total, 62 filaments were measured from 50 micrographs.

#### Model building

Initial atomic models were generated using AlphaFold2 and ColabFold. ^26,58^ These models were positioned as rigid bodies into the cryo-EM density map using Coot^61^ and optimized to fit the density using real-space refinement with torsion, planar peptide and Ramachandran restraints applied. *Trans* peptide restraints were turned off when modeling *cis*-Prolines. The PPII helix was built *de novo*, and the prolines converted to hydroxyproline. *N*- and *O*-linked glycans were built from individual saccharide moieties and linked to the protein sidechains. One complete MST1 monomer was built first and then used to model an antiparallel copy. The conformations of the two copies are not identical with a shift in the positions of Ig-like domains D5–7 of up to 6 Å. The density for MST3 was interpreted with a model of residues 1453–1628, corresponding to the first two of its 42 proline-rich repeats. The final atomic model was refined against the C1 map using real-space refinement in Phenix^69^ with Ramachandran and rotamer restraints applied. Restraints for the saccharides were generated using Phenix.elbow.^70^ Model statistics were calculated using MolProbity implemented in Phenix^68^ and reported in Table S1.

#### MST3 identification

MST3 was identified from the cryo-EM density based on the presence of glycans surrounding its PPII helix. We assumed that every glycosylated residue was a hydroxyproline. This analysis revealed two alternating segments of density: one with 13 glycosylated hydroxyproline residues and one with 11 glycosylated hydroxyproline residues. The shorter stretch of continuous hydroxyproline residues was followed by a region with a distinctive glycosylation pattern that we interpreted as corresponding to glycosylated hydroxyproline residues separated by two non-proline residues. Based on this information, we searched a .fasta file of v.6.1 of the *C. reinhardtii* proteome^53^ downloaded from Phytozome^80^ for instances of proteins with P_13_Y_n<20_P_11_(XXP)_3_ motifs, where P=proline, Y=any residue, and X=any residue other than proline. Proteins with fewer than 2,500 residues were discounted based on the assumption that the stalk was a single protein capable of spanning the 800-nm length of the mastigoneme. This identified Cre06.g309951 (MST3) as containing 42 copies of the P_13_Y_n<20_P_11_(XXP)_3_ motif.

The sequence annotation of Cre06.g309951 in Phytozome varies between versions 5.6^54^ and 6.1 of the *C. reinhardtii* proteome. The annotated version of the sequence in v.5.6 contains a longer N terminus that encodes a signal peptide, a poly(proline)-rich region and five C-type lectin domains. As our mass spectrometry analysis of purified mastigonemes identified a peptide mapping to this extended N terminus (Table S3), we appended the N terminus (residues 1–1,183) of v.5.6 onto the v.6.1 sequence, creating a protein that is 8,572 residues in length. Numbering for this chimera is used throughout the manuscript. Two of the *mst3* mutants (*mst3–1* and *mst3–2*) that we validated to be lacking mastigonemes (Figure S5) are created by insertions in exon 3 (Figure S4B) that is only present in the longer construct, providing further confidence in the revised annotation.

#### Bioinformatics

Protein sequences were obtained from Phytozome.^80^ Protein sequence alignment and cladogram construction was performed with ClustalOmega.^62^ Signal peptides and their cleavage sites were identified using SignalP-6.0.^27^ Transmembrane helices were identified using DeepTMHMM.^64^ Domains were identified from the primary sequence using InterProScan.^67^ Structurally similar proteins were identified from PDB files using DALI server^63^ and FoldSeek.^66^ Predictions of SIP complexes were obtained using AlphaFold Multimer.^57^ All figures of cryo-EM maps or atomic models were generated using ChimeraX v.1.5.^60^ Topology diagrams were drawn using PDBSum.^71^ Structural biology software were installed and managed by SBGrid.^73^ Mass spectrometry data were analyzed and plotted using Prism v.9.5.0 (GraphPad) and JMP Pro v.16 (JMP).

#### Quantification and statistical analysis

Estimations of the resolution of the cryo-EM density maps are based on the 0.143 FSC criterion.^81^ All statistical validation performed on the atomic models was done using Phenix and MolProbity^68^ (Table S1). Statistical analysis of swimming speed differences (Figure S5C) was determined using an unpaired t test with Welch’s correction. Significance in the analysis of the multiplexed quantitative mass spectrometry data was determined using multiple unpaired t tests.

## Supplementary Material

1 Figure S1. Cryo-EM processing of the mastigoneme, related to Figures 1 and 2
(A) Scheme of the initial processing steps starting from a motion-corrected micrograph of mastigonemes among doublet microtubules. Mastigonemes were selected from the micrographs using Filament Tracer in CryoSPARC. Contaminating doublet microtubules were excluded during two-dimensional classification (classes marked with a green dot correspond to mastigoneme projections). After *ab initio* reconstruction and homogeneous refinement, D1 symmetry was identified and applied. Following helical reconstruction and symmetry expansion, local refinement using short cylindrical masks was used to improve the resolution, with the middle section reaching a nominal resolution of 3.0 Å based on the Fourier Shell Correlation (FSC) = 0.143 criterion.
(B) Detail of the subsequent processing scheme, which reverted to C1 symmetry after identifying a second component of the mastigoneme that did not follow the same helical symmetry as MST1. As in (A), local refinement with short cylindrical masks was used to improve local map quality with resolutions reaching 3.1 Å.

2 Figure S2. AlphaFold2 modeling of MST1 and MST2, related to Figure 1
(A) Cladogram showing the hypothesized evolutionary relationship between MST1-like proteins from Chlorophyceae species. The cladogram was constructed by aligning protein sequences with ClustalOmega.
(B) Sequence coverage for MST1 identified using ColabFold. Almost all of the sequences identified align to the first repeats in the cysteine-rich domain, leading to exceptionally low coverage for the rest of the protein.
(C) AlphaFold2 prediction of MST1 colored by domain. Most of the immunoglobulin-like domains are not or incorrectly predicted.
(D) A predicted aligned error (PAE) plot showing the low confidence in the prediction of MST1.
(E) A predicted local distance difference test (pLDDT) plot showing per-residue confidence. Most regions fall below 60 and are of low confidence. Only parts of the cysteine-rich domain are built with a confidence that would be classed as “accurate.”
(F) An example of the improved AlphaFold2 prediction achieved by predicting individual domains, in this case, immunoglobulin-like domain D1.
(G) Sequence coverage for MST1 D1 identified using ColabFold. Good coverage is achieved except for the first 40 residues, which likely correspond to a cleaved signal peptide.
(H) PAE plot showing the confidence in the predicted model of the D1 domain.
(I) Domain architecture of MST2. Compared with MST1 (Figure 1E), MST2 lacks the nine N-terminal immunoglobulin domains but still has a predicted signal peptide (SP) cleavage site, a cysteine-rich domain, and a C-terminal poly(proline) [p(P)] region.
(J) Homology model of MST2 colored by domain. Loop insertions absent in MST1 are colored red.
(K) Model of an MST2 polymer based on the structure of the MST1-containing mastigoneme. The interfaces that form the antiparallel homodimer and the connection between dimers within a strand are conserved and devoid of insertions.
(L) Sequence alignment of the poly(proline)-rich regions of *C. reinhardtii* MST1 and MST2. Identical residues are marked with an asterisk.

3 Figure S3. N-glycosylation of MST1, related to Figure 3
(A) An atomic model of MST1 showing the positions of three asparagine residues (indicated with red spheres) confirmed to be *N*-glycosylated based on the cryo-EM density. An asparagine residue within the poly(hydroxyproline) helix incorrectly predicted to be *N*-glycosylated is indicated with a blue sphere. Density maps of each site are shown in (B)–(E).
(B) Cryo-EM density supporting the modeling of two *N*-Acetylglucosamine (NAG) moieties attached to N851 in the D7 immunoglobulin-like domain. This residue is not conserved in other Chlorophyceae species.
(C) Cryo-EM density supporting at least three saccharide moieties attached to N1194 in the cysteine-rich domain. The first two saccharide units have been modeled as *N*-acetylglucosamine, the third marked with an asterisk. Below shows the sequence conservation of the *N*-glycosylation recognition site in other species.
(D) Cryo-EM density supporting at least six saccharide moieties attached to N1643 in the cysteine-rich domain. This site is conserved across *Chlorophyceae* species.
(E) Density for N1924 in the poly(proline)-rich region. Despite occurring in a recognition site predicted to be *N*-glycosylated, N1924 is unmodified.

4 Figure S4. Purification of native *C. reinhardtii* mastigonemes and validation of CLiP mutant strains, related to Figures 5 and 7
(A) Silver-stained sodium dodecyl sulfate-polyacrylamide gel electrophoresis (SDS-PAGE) gel showing a band corresponding to MST1. The apparent molecular weight of MST1 is higher than its predicted molecular mass due to the presence of glycans.
(B) Negative-stain electron micrograph showing multiple purified mastigonemes.
(C) A magnified negative-stain image of a single mastigoneme showing the transition of the mastigoneme into a thin-end filament.
(D) Schematic representation of the MST3, PKD2, MST1, and SIP genes. Primer pairs flanking the insertion sites in each CLiP mutant are shown. Gray arrows indicate 5′ and 3′ untranslated regions. Colored arrows indicate exons.
(E) Gel electrophoresis analysis of PCR products amplified from wild-type and *mst3* mutant genomic DNA. PCR products amplified from wild-type DNA yield the expected size band (indicated by black arrowheads), while PCR products from the mutant strains (indicated by white arrowheads) are larger due to the presence of the insertion.
(F) Gel electrophoresis analysis of PCR products amplified from wild-type and *pkd2*, *mst1*, and *sip* mutant genomic DNA. PCR products amplified from wild-type DNA yield the expected size band (indicated with black arrowheads), while PCR products from the *pkd2* and *mst1* mutants (white arrowheads) are larger due to the presence of the insertion. No band was detected for the *sip* mutant.

5 Figure S5. *C. reinhardtii mst1, pkd2, sip*, and *mst3* mutants lack mastigonemes, related to Figure 7
(A) Negative-stain electron microscopy (EM) was used to compare *C. reinhardtii mst1, pkd2*, and *sip* mutant strains from the CLiP collection with the wild-type parental strain (CC-5325). For each strain, an overview micrograph is shown (top), with a yellow box highlighting the region viewed in the higher magnification image (below). Only cilia of the wild-type parental strain displays mastigonemes.
(B) Negative-stain EM of four mst3 mutants (numbered 1–4). For each strain, an overview micrograph is shown together with a composite of negative-stain micrographs along the length of a single cilium. One micrograph from each composite is highlighted with a yellow box and shown in (B).
(C) Negative-stain micrographs showing the boxed micrographs from (B). All strains lack mastigonemes.

6 Figure S6. Characterization of *C. reinhardtii mst1, pkd2, sip*, and *mst3* mutants, related to Figure 7
(A) Raincloud plots comparing the swimming velocity (μm/s) of four mst3 mutants with the wild-type (WT CC-5325) parental strain. Each point represents an individual measurement (n = 237 for WT, n = 260 for *mst3*-*1*, n = 188 for *mst3–2*, n = 341 for *mst3–3*, and n = 125 for *mst3–4*). In the box plot, the central dashed line represents the median and the solid line the mean. The bottom and top of the box representing the first (Q1) and third (Q3) quartiles, respectively. The whiskers extend to the highest and the lowest scores. Significance (p < 0.0001) was determined using an unpaired Welch t test.
(B) Volcano plots generated from quantitative mass spectrometry data of isolated cilia. Each plot shows significance versus enrichment for a *C. reinhardtii* mutant strain compared with wild type (WT CC-5325). Individual proteins are shown as gray dots except for the predicted proteins of the mastigoneme (colored) and MST2 and predicted SIP binders (black). The high number of enriched proteins in the *mst1* mutant are contaminants from the cell body not found in the WT strain.

7 Figure S7. Identification of potential SIP binders, related to Figure 7
(A) Predicted domain architecture of seven proteins identified in v.6.1 of the *C. reinhardtii* proteome as having partial PKD2-like domains. Domains were identified from primary sequence using InterProScan^67^ and from AlphaFold2 predictions using FoldSeek.^66^ Globular domains predicted by AlphaFold2 that could not be confidently classified are labeled as “Unknown.” The amino acid length of each protein is indicated at the C terminus. Abbreviations: LRR, leucine-rich repeat; SRCR, scavenger receptor cysteine-rich; SEA, sea urchin sperm protein, enterokinase, agrin; cysteine-rich, small cysteine-rich domain. All except Cre11.g479383 have poly(proline)-rich regions.
(B) AlphaFold Multimer models (top) and PAE plots (bottom) for the PKD2-like domain of each protein listed in (A) in complex with SIP. In each case, SIP completes the PKD2-like fold by contributing a transmembrane helix to the voltage-sensor-like domain and two helices and a β-strand to the TOP domain. For some proteins, long flexible loops between domains have been removed for clarity.

MMC1

## Figures and Tables

**KEY RESOURCES TABLE:** 

REAGENT or RESOURCE	SOURCE	IDENTIFIER

Antibodies		

Rabbit polyclonal anti-PKD2-Cter	Huang et al.^48^	N/A
Rabbit polyclonal anti-PKD2-loop	Huang et al.^48^	N/A
Rabbit polyclonal anti-MST1	Das et al.^19^	N/A
Rabbit polyclonal anti-SIP	Das et al.^19^	N/A
Rabbit polyclonal anti-β-tubulin	Cell Signaling Technology	RRID: AB_2210545
Donkey polyclonal anti-rabbit-IgG, horseradish peroxidase conjugated	Sigma-Aldrich	RRID: AB_92591

Chemicals, peptides, and recombinant proteins		

Dibucaine hydrochloride	Millipore Sigma	Cat. #D0638
DreamTaq DNA polymerase	Thermo Scientific	Cat. #EP0701
Hydroxylamine	Sigma Aldrich	Cat. #467804-10ML
Q5 High-fidelity DNA polymerase	New England Biolabs	Cat #M0491S
QuickExtract DNA extraction solution	Biosearch Technologies	Cat. #QE09050
Lysyl Endopeptidase (Lys-C)	Wako	Cat. #129-02541
Sera-Mag magnetic carboxylate modified particles (Hydrophobic)	Cytiva	Cat. #44152105050250
Sera-Mag^™^ Magnetic carboxylate modified particles (Hydrophylic)	Cytiva	Cat. #24152105050250
SurePAGE Bis-Tris gel, 4–20%, 15 wells	GenScript	Cat. #M00656
SyproRuby	Sigma-Aldrich	Cat. #S4942
TMTpro isobaric reagents	Thermo Scientific	Cat. #A34808
Tris-MOPS-SDS Running Buffer Powder	GenScript	Cat. #M00138
Trypsin	Promega	Cat. #V5113

Critical commercial assays		

ECL Prime Western Blotting Detection Reagent	Cytiva	Cat. #RPN2236
High pH Reversed-Phase Benchtop Fractionation Kit	Thermo Scientific	Cat. #84868

Deposited data		

*Chlamydomonas reinhardtii* mastigoneme (constituent map 1)	This study	EMDB: EMD-43889
*Chlamydomonas reinhardtii* mastigoneme (constituent map 2)	This study	EMDB: EMD-43890
Chlamydomonas reinhardtii mastigoneme (constituent map 3)	This study	EMDB: EMD-43891
Composite cryo-EM map of the *C. reinhardtii* mastigoneme	This study	EMDB: EMD-43892
Atomic model of the *C. reinhardtii* mastigoneme	This study	PDB: 9B4H
*C. reinhardtii* CC-4532 v6.1 proteome	Craig et al.^53^	https://phytozome-next.jgi.doe.gov/info/CreinhardtiiCC_4532_v6_1
*C. reinhardtii* v5.6 proteome	Merchant et al.^54^	https://phytozome-next.jgi.doe.gov/info/Creinhardtii_v5_6
*C. reinhardtii mst3* strain swimming speed analysis (raw images)	This study	https://zenodo.org/records/10627574
Comparative analysis of *C. reinhardtii* cilia from mastigoneme mutants	This study	PRIDE accession: PXD049250

Experimental models: Organisms/strains		

*Chlamydomonas reinhardtii* (CC-1690)	Chlamydomonas Resource Center	https://www.chlamycollection.org
*Chlamydomonas reinhardtii agg1, nit1, nit2* (CC-4402)	Chlamydomonas Resource Center	https://www.chlamycollection.org
*Chlamydomonas reinhardtii cw15* (CC-5325)	Li et al.^55^	https://www.chlamycollection.org
*Chlamydomonas reinhardtii mst3-1* (LMJ.RY0402.079427)	Li et al.^47^	https://www.chlamycollection.org
*Chlamydomonas reinhardtii mst3-2* (LMJ.RY0402.104433)	Li et al.^47^	https://www.chlamycollection.org
*Chlamydomonas reinhardtii mst3-3* (LMJ.RY0402.120927)	Li et al.^47^	https://www.chlamycollection.org
*Chlamydomonas reinhardtii mst3-4* (LMJ.RY0402.226217)	Li et al.^47^	https://www.chlamycollection.org
*Chlamydomonas reinhardtii sip* (LMJ.RY0402.143879)	Li et al.^47^	https://www.chlamycollection.org
*Chlamydomonas reinhardtii mst1* (LMJ.RY0402.052413)	Li et al.^47^	https://www.chlamycollection.org
*Chlamydomonas reinhardtii pkd2* (LMJ.RY0402.204581)	Li et al.^47^	https://www.chlamycollection.org

Software and algorithms		

μMANAGER	Edelstein et al.^56^	https://micro-manager.org/
AlphaFold2	Jumper et al.^26^	
AlphaFold Multimer	Evans et al.^57^	
ColabFold	Mirdita et al.^58^	https://github.com/sokrypton/ColabFold
Comet v2019.01 rev. 5	Eng et al.^59^	
ChimeraX v1.5	Goddard et al.^60^	https://www.cgl.ucsf.edu/chimerax/
Coot v0.9.8.5	Brown et al.^61^	https://www2.mrc-lmb.cam.ac.uk/personal/pemsley/coot/
ClustalOmega	Sievers et al.^62^	https://www.ebi.ac.uk/Tools/msa/clustalo/
CryoSPARC	Punjani et al.^24^	https://cryosparc.com/
CryoSPARC Non-uniform refinement	Punjani et al^25^	https://cryosparc.com/
DALI server	Holm, 2022^63^	http://ekhidna2.biocenter.helsinki.fi/dali/
DeepTMHMM	Hallgren et al^64^	https://dtu.biolib.com/DeepTMHMM
Fiji	Schindelin et al.^65^	https://imagej.net/software/fiji/
FoldSeek	Kempen et al.^66^	https://search.foldseek.com/search
GFY Core platform	Harvard University	https://gygi.hms.harvard.edu/software.html
Image Lab	Bio-Rad	https://www.bio-rad.com/en-us/product/image-lab-software?ID=KRE6P5E8Z
InterProScan	Jones et al.^67^	https://www.ebi.ac.uk/interpro/
JMP Pro v.16.	JMP	https://www.jmp.com
MolProbity	Williams et al.^68^	http://molprobity.biochem.duke.edu/
NEB Tm Calculator v1.16.5	New England Biolabs	https://tmcalculator.neb.com/#!/main
Phenix.real_space_refine	Afonine et al.^69^	https://phenix-online.org/
Phenix.elbow	Moriarty et al.^70^	https://phenix-online.org/documentation/reference/elbow_gui.html
PDBSum	Laskowski et al.^71^	https://www.ebi.ac.uk/thornton-srv/databases/pdbsum/Generate.html
Prism v.9.5.0	GraphPad	https://www.graphpad.com/
relion_image_handler	Scheres,^72^	https://www3.mrc-lmb.cam.ac.uk/relion
SBGrid	Morin et al.^73^	https://sbgrid.org/
Sequest	Thermo Fisher Scientific	
SignalP-6.0	Teufel et al.^27^	https://services.healthtech.dtu.dk/services/SignalP-6.0/

Other		

Carbon-coated copper grids, 200 mesh	Electron Microscopy Sciences	Cat. #CF200-Cu

## Highlights

- Cryo-EM reveals the composition and structure of an algal mastigoneme filament
- MST3 anchors mastigonemes to ciliary membranes via a PKD-like domain aided by SIP
- *O*-linked hydroxyproline glycosylation sheaths PPII helices in a carbohydrate casing
- Serine galactosylation hydrogen bonds with the poly(hydroxyproline) backbone
