# Supplementary figures and images for: Mastigoneme structure reveals insights into the O-linked glycosylation code of native hydroxyproline-rich helices
Source: Cell. Author manuscript; Available in PMC 2024 Apr 14. (PMC11015965; doi:10.1016/j.cell.2024.03.005)

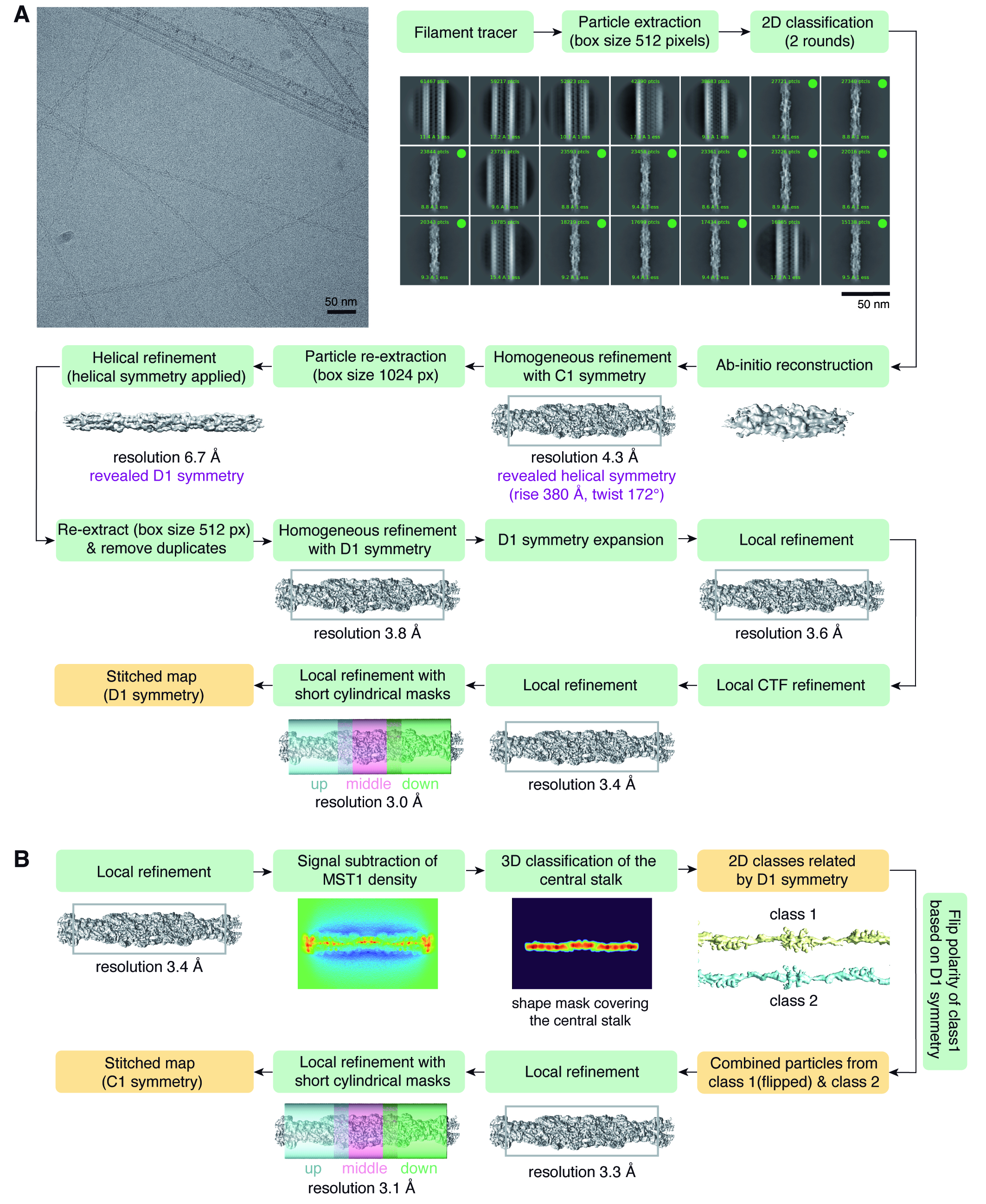

Supplement: 1 — Figure S1. Cryo-EM processing of the mastigoneme, related to Figures 1 and 2 (A) Scheme of the initial processing steps starting from a motion-corrected micrograph of mastigonemes among doublet microtubules. Mastigonemes were selected from the micrographs using Filament Tracer in CryoSPARC. Contaminating doublet microtubules were excluded during two-dimensional classification (classes marked with a green dot correspond to mastigoneme projections). After ab initio reconstruction and homogeneous refinement, D1 symmetry was identified and applied. Following helical reconstruction and symmetry expansion, local refinement using short cylindrical masks was used to improve the resolution, with the middle section reaching a nominal resolution of 3.0 Å based on the Fourier Shell Correlation (FSC) = 0.143 criterion. (B) Detail of the subsequent processing scheme, which reverted to C1 symmetry after identifying a second component of the mastigoneme that did not follow the same helical symmetry as MST1. As in (A), local refinement with short cylindrical masks was used to improve local map quality with resolutions reaching 3.1 Å. [file NIHMS1977199-supplement-1.jpg]

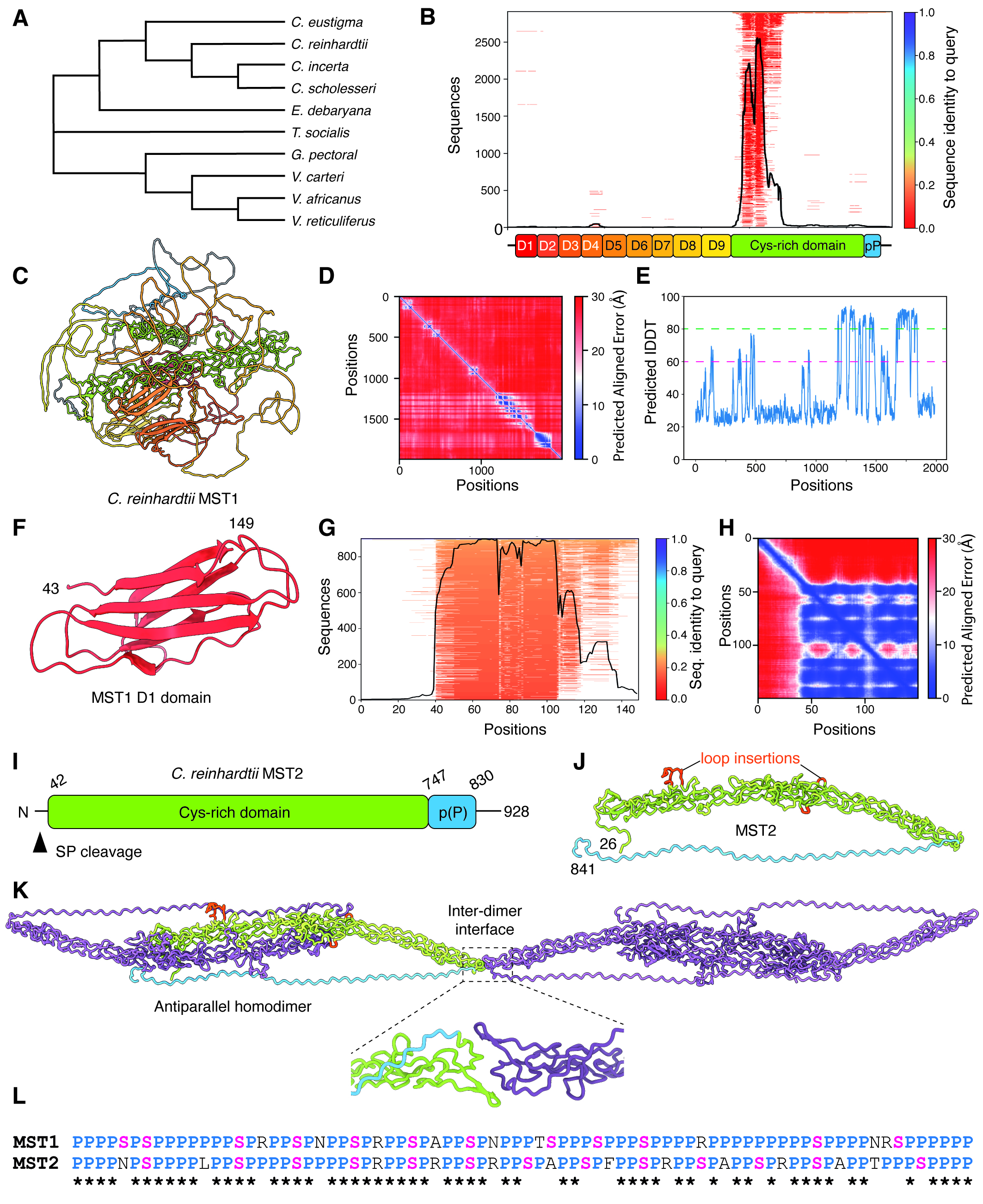

Supplement: 2 — Figure S2. AlphaFold2 modeling of MST1 and MST2, related to Figure 1 (A) Cladogram showing the hypothesized evolutionary relationship between MST1-like proteins from Chlorophyceae species. The cladogram was constructed by aligning protein sequences with ClustalOmega. (B) Sequence coverage for MST1 identified using ColabFold. Almost all of the sequences identified align to the first repeats in the cysteine-rich domain, leading to exceptionally low coverage for the rest of the protein. (C) AlphaFold2 prediction of MST1 colored by domain. Most of the immunoglobulin-like domains are not or incorrectly predicted. (D) A predicted aligned error (PAE) plot showing the low confidence in the prediction of MST1. (E) A predicted local distance difference test (pLDDT) plot showing per-residue confidence. Most regions fall below 60 and are of low confidence. Only parts of the cysteine-rich domain are built with a confidence that would be classed as “accurate.” (F) An example of the improved AlphaFold2 prediction achieved by predicting individual domains, in this case, immunoglobulin-like domain D1. (G) Sequence coverage for MST1 D1 identified using ColabFold. Good coverage is achieved except for the first 40 residues, which likely correspond to a cleaved signal peptide. (H) PAE plot showing the confidence in the predicted model of the D1 domain. (I) Domain architecture of MST2. Compared with MST1 (Figure 1E), MST2 lacks the nine N-terminal immunoglobulin domains but still has a predicted signal peptide (SP) cleavage site, a cysteine-rich domain, and a C-terminal poly(proline) [p(P)] region. (J) Homology model of MST2 colored by domain. Loop insertions absent in MST1 are colored red. (K) Model of an MST2 polymer based on the structure of the MST1-containing mastigoneme. The interfaces that form the antiparallel homodimer and the connection between dimers within a strand are conserved and devoid of insertions. (L) Sequence alignment of the poly(proline)-rich regions of C. reinhardt [file NIHMS1977199-supplement-2.jpg]

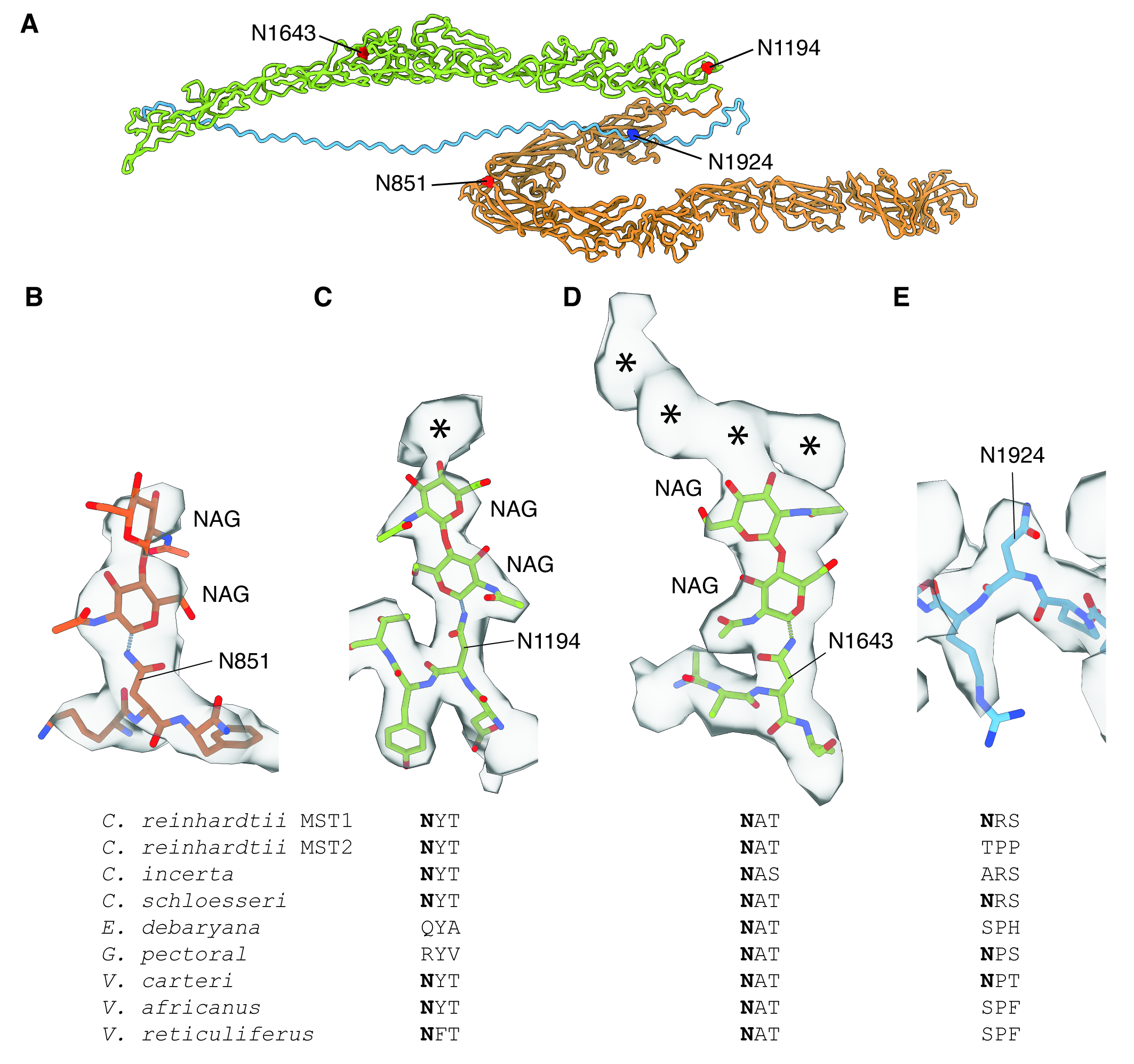

Supplement: 3 — Figure S3. N-glycosylation of MST1, related to Figure 3 (A) An atomic model of MST1 showing the positions of three asparagine residues (indicated with red spheres) confirmed to be N-glycosylated based on the cryo-EM density. An asparagine residue within the poly(hydroxyproline) helix incorrectly predicted to be N-glycosylated is indicated with a blue sphere. Density maps of each site are shown in (B)–(E). (B) Cryo-EM density supporting the modeling of two N-Acetylglucosamine (NAG) moieties attached to N851 in the D7 immunoglobulin-like domain. This residue is not conserved in other Chlorophyceae species. (C) Cryo-EM density supporting at least three saccharide moieties attached to N1194 in the cysteine-rich domain. The first two saccharide units have been modeled as N-acetylglucosamine, the third marked with an asterisk. Below shows the sequence conservation of the N-glycosylation recognition site in other species. (D) Cryo-EM density supporting at least six saccharide moieties attached to N1643 in the cysteine-rich domain. This site is conserved across Chlorophyceae species. (E) Density for N1924 in the poly(proline)-rich region. Despite occurring in a recognition site predicted to be N-glycosylated, N1924 is unmodified. [file NIHMS1977199-supplement-3.jpg]

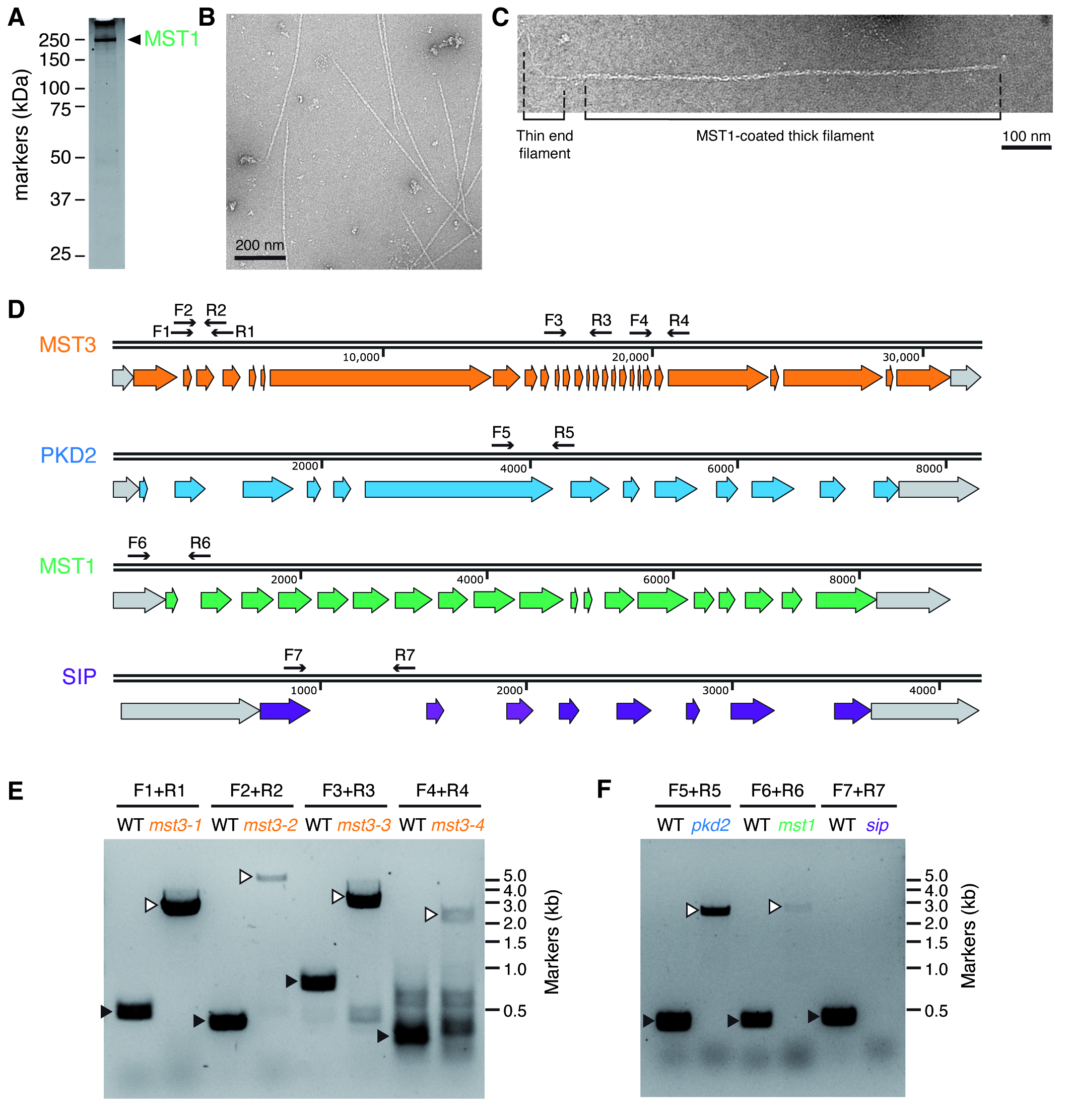

Supplement: 4 — Figure S4. Purification of native C. reinhardtii mastigonemes and validation of CLiP mutant strains, related to Figures 5 and 7 (A) Silver-stained sodium dodecyl sulfate-polyacrylamide gel electrophoresis (SDS-PAGE) gel showing a band corresponding to MST1. The apparent molecular weight of MST1 is higher than its predicted molecular mass due to the presence of glycans. (B) Negative-stain electron micrograph showing multiple purified mastigonemes. (C) A magnified negative-stain image of a single mastigoneme showing the transition of the mastigoneme into a thin-end filament. (D) Schematic representation of the MST3, PKD2, MST1, and SIP genes. Primer pairs flanking the insertion sites in each CLiP mutant are shown. Gray arrows indicate 5′ and 3′ untranslated regions. Colored arrows indicate exons. (E) Gel electrophoresis analysis of PCR products amplified from wild-type and mst3 mutant genomic DNA. PCR products amplified from wild-type DNA yield the expected size band (indicated by black arrowheads), while PCR products from the mutant strains (indicated by white arrowheads) are larger due to the presence of the insertion. (F) Gel electrophoresis analysis of PCR products amplified from wild-type and pkd2, mst1, and sip mutant genomic DNA. PCR products amplified from wild-type DNA yield the expected size band (indicated with black arrowheads), while PCR products from the pkd2 and mst1 mutants (white arrowheads) are larger due to the presence of the insertion. No band was detected for the sip mutant. [file NIHMS1977199-supplement-4.jpg]

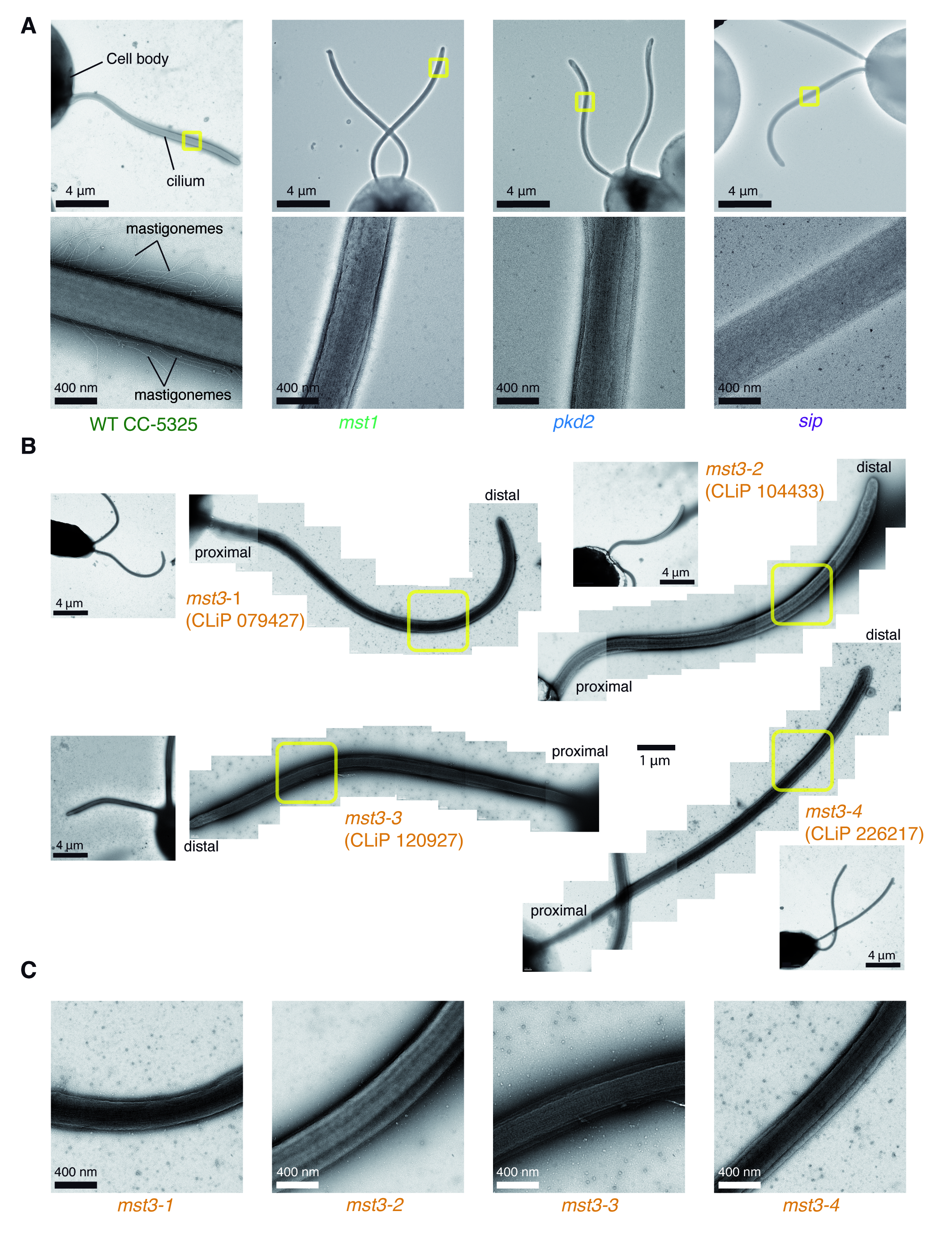

Supplement: 5 — Figure S5. C. reinhardtii mst1, pkd2, sip, and mst3 mutants lack mastigonemes, related to Figure 7 (A) Negative-stain electron microscopy (EM) was used to compare C. reinhardtii mst1, pkd2, and sip mutant strains from the CLiP collection with the wild-type parental strain (CC-5325). For each strain, an overview micrograph is shown (top), with a yellow box highlighting the region viewed in the higher magnification image (below). Only cilia of the wild-type parental strain displays mastigonemes. (B) Negative-stain EM of four mst3 mutants (numbered 1–4). For each strain, an overview micrograph is shown together with a composite of negative-stain micrographs along the length of a single cilium. One micrograph from each composite is highlighted with a yellow box and shown in (B). (C) Negative-stain micrographs showing the boxed micrographs from (B). All strains lack mastigonemes. [file NIHMS1977199-supplement-5.jpg]

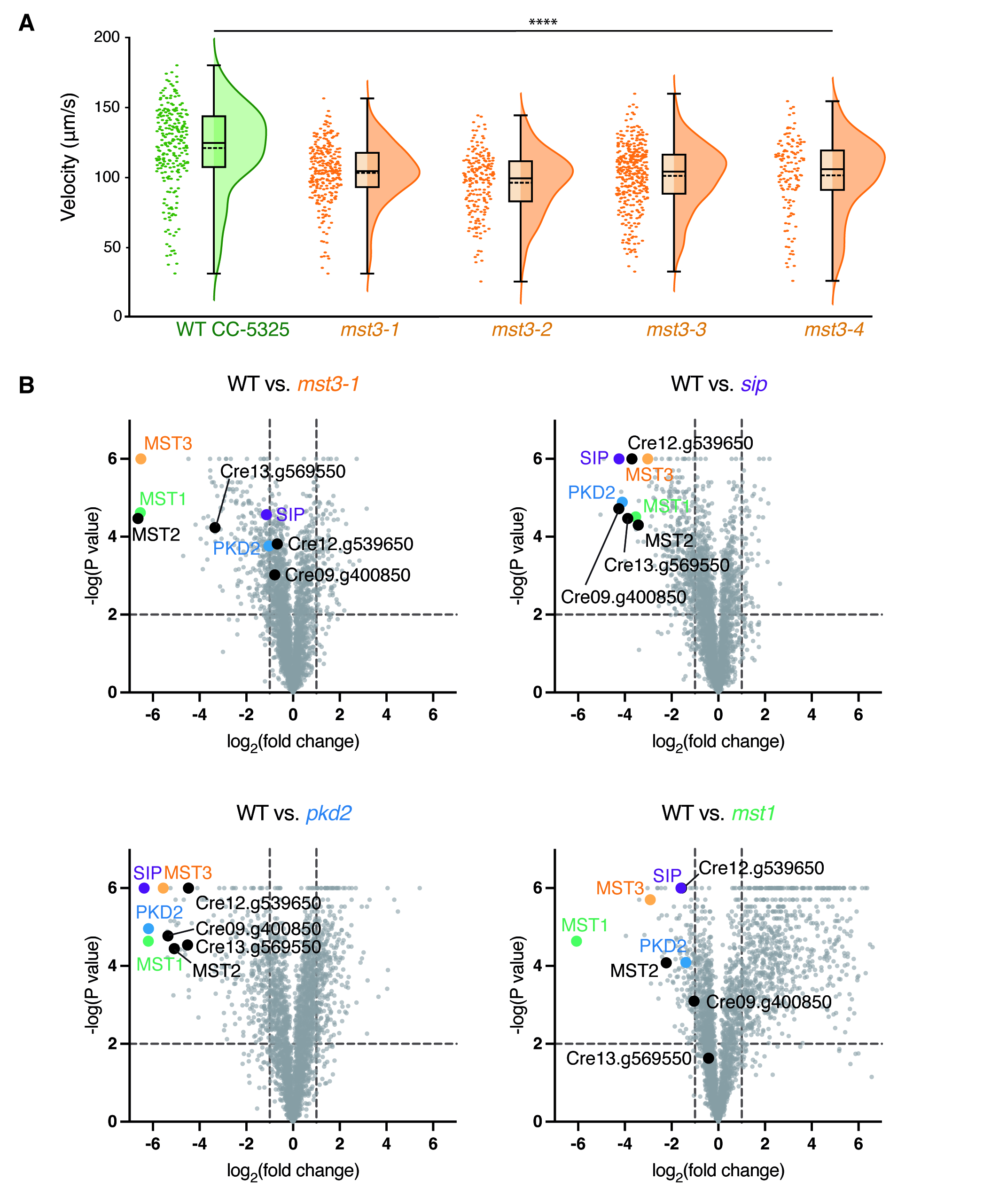

Supplement: 6 — Figure S6. Characterization of C. reinhardtii mst1, pkd2, sip, and mst3 mutants, related to Figure 7 (A) Raincloud plots comparing the swimming velocity (μm/s) of four mst3 mutants with the wild-type (WT CC-5325) parental strain. Each point represents an individual measurement (n = 237 for WT, n = 260 for mst3-1, n = 188 for mst3–2, n = 341 for mst3–3, and n = 125 for mst3–4). In the box plot, the central dashed line represents the median and the solid line the mean. The bottom and top of the box representing the first (Q1) and third (Q3) quartiles, respectively. The whiskers extend to the highest and the lowest scores. Significance (p < 0.0001) was determined using an unpaired Welch t test. (B) Volcano plots generated from quantitative mass spectrometry data of isolated cilia. Each plot shows significance versus enrichment for a C. reinhardtii mutant strain compared with wild type (WT CC-5325). Individual proteins are shown as gray dots except for the predicted proteins of the mastigoneme (colored) and MST2 and predicted SIP binders (black). The high number of enriched proteins in the mst1 mutant are contaminants from the cell body not found in the WT strain. [file NIHMS1977199-supplement-6.jpg]

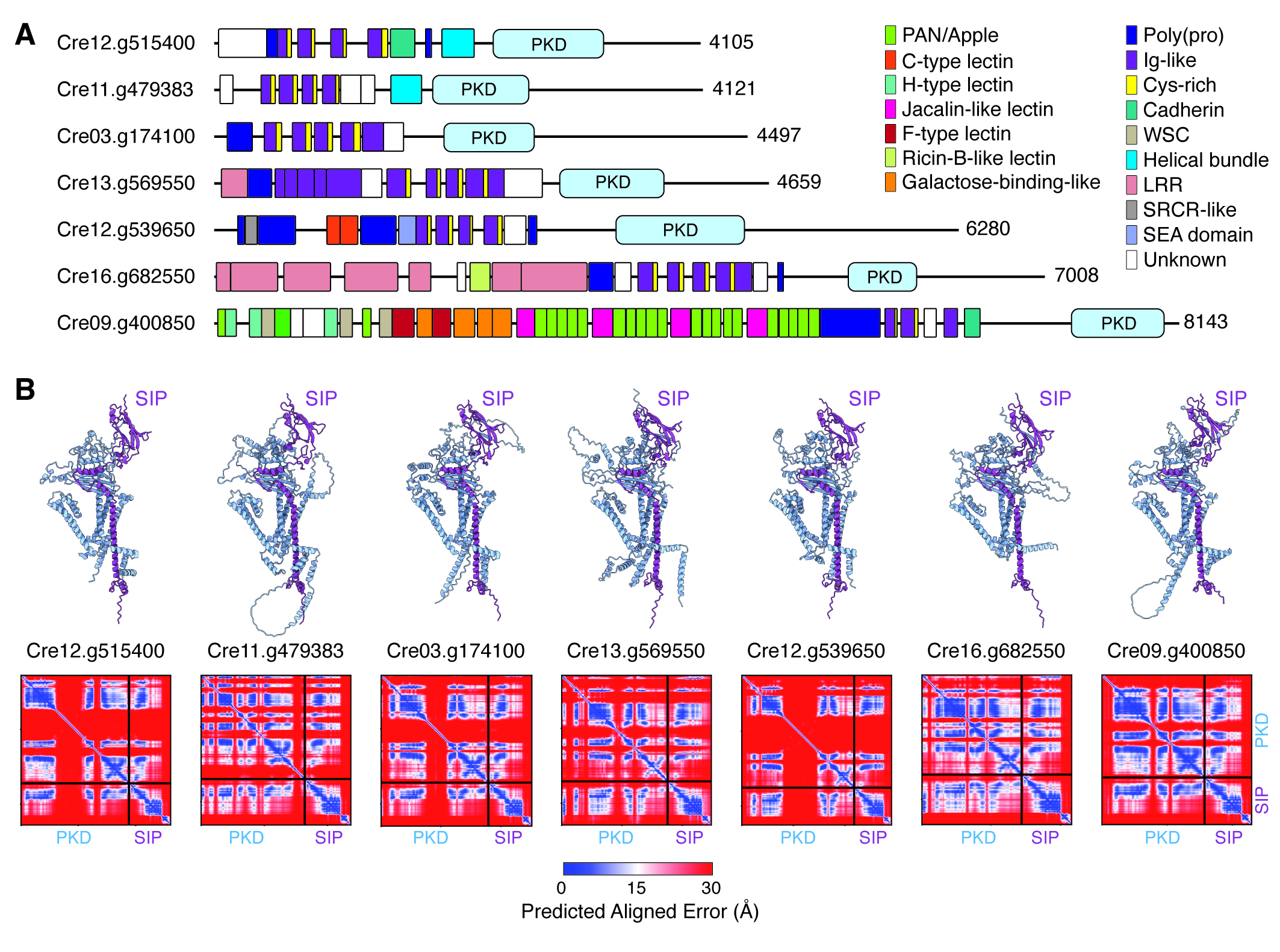

Supplement: 7 — Figure S7. Identification of potential SIP binders, related to Figure 7 (A) Predicted domain architecture of seven proteins identified in v.6.1 of the C. reinhardtii proteome as having partial PKD2-like domains. Domains were identified from primary sequence using InterProScan67 and from AlphaFold2 predictions using FoldSeek.66 Globular domains predicted by AlphaFold2 that could not be confidently classified are labeled as “Unknown.” The amino acid length of each protein is indicated at the C terminus. Abbreviations: LRR, leucine-rich repeat; SRCR, scavenger receptor cysteine-rich; SEA, sea urchin sperm protein, enterokinase, agrin; cysteine-rich, small cysteine-rich domain. All except Cre11.g479383 have poly(proline)-rich regions. (B) AlphaFold Multimer models (top) and PAE plots (bottom) for the PKD2-like domain of each protein listed in (A) in complex with SIP. In each case, SIP completes the PKD2-like fold by contributing a transmembrane helix to the voltage-sensor-like domain and two helices and a β-strand to the TOP domain. For some proteins, long flexible loops between domains have been removed for clarity. [file NIHMS1977199-supplement-7.jpg]
