# Supplementary material for: Mastigoneme structure reveals insights into the O-linked glycosylation code of native hydroxyproline-rich helices
Source: Cell. Author manuscript; Available in PMC 2024 Apr 14. (PMC11015965; doi:10.1016/j.cell.2024.03.005)
Supplement: MMC1 [file NIHMS1977199-supplement-MMC1.pdf]

Cell, Volume *187*

Supplemental information

Mastigoneme structure reveals insights into the *O*-linked glycosylation code of native hydroxyproline-rich helices

Jin Dai, Meisheng Ma, Qingwei Niu, Robyn J. Eisert, Xiangli Wang, Poulomi Das, Karl F. Lechtreck, Susan K. Dutcher, Rui Zhang, and Alan Brown

SUPPLEMENTARY TABLES

Supplementary Table 1. Cryo-EM processing, refinement, and validation statistics. Data collection statistics are provided in Reference [S1]. Related to Figure 1.

	C. reinhardtii mastigoneme (EMDB: EMD-43892) (PDB: 9B4H)	Mastigoneme distal tip
**Data processing**		
No. of movies	19,601	1,303
Pixel size (Å)	1.39[a]	1.39
Picking method	CryoSPARC filament tracer	Manual
Initial Model	Ab-initio reconstruction	Ab-initio reconstruction
Refinement method(s)	Homogeneous refinement, helical refinement, non-uniform refinement	Helical refinement
Symmetry imposed	C1	C1
Initial particle images (no.)	735,338[b]	14,758
Final particle images (no.)	687,452	4,015
Map resolution (Å)	3.1 Å	13.3 Å
**Refinement**		
Initial model used	AlphaFold2 predictions; de novo modeling	
Model composition[c]		
Protein chains	3 (2 copies of MST1, one copy of MST3)	
Non-H atoms	32328	
Protein residues	3925	
Saccharides	380	
Correlation coefficient (CCmask)	0.88	
R.M.S. deviations		
Bond lengths (Å)	0.003	
Bond angles (°)	0.732	
Validation		
MolProbity score	2.2	
Clashscore	11.0	
Poor rotamers (%)	3.2	
Ramachandran		
Favored (%)	96.7	
Allowed (%)	3.3	
Outliers (%)	0	

[a] Micrographs come from data collected on two different Krios microscopes with different pixel sizes. Data collected on the CWRU Krios (Å/pixel = 1.34) were rescaled to the pixel size of the WU Krios data (Å/pixel = 1.39).

[b] Number of particles from filament tracing and particle extraction using 70 Å step size, after removing non-mastigoneme particles using 2D classification.

[c] The deposited model is the minimum required to regenerate the complete filament using helical symmetry

Supplementary Table 2. Mass spectrometry analysis of purified mastigonemes. Proteins with fewer than one peptide were excluded from the analysis. Related to Figure 5 and Figure S4.

Gene Symbol	Phytozome ID	MW (kDa)	Unique	Total	AVG
MST1	Cre16.g650600_4532.1.p	205.16	40	71	3.8915
MST3	Cre06.g309951_4532.1.p	866.21	9	11	3.6214
FMG1B	Cre09.g392867_4532.1.p	447.74	40	58	3.8353
EEF1A3	Cre06.g263450_4532.1.p	50.79	11	15	3.0441
TUA1	Cre03.g190950_4532.1.p	49.55	11	12	3.4677
TUB2	Cre12.g549550_4532.1.p	49.59	9	15	2.9472
DHC15	Cre11.g476050_4532.1.p	513.51	8	8	2.2563
IDA5	Cre13.g603700_4532.1.p	41.81	7	15	2.9816
CWP2	Cre06.g258800_4532.1.p	131.04	7	9	2.5335
FAP325	Cre06.g263250_4532.1.p	366.51	6	9	5.3195
FAP237	Cre06.g278170_4532.1.p	133.14	5	5	3.6547
FAP113	Cre07.g321400_4532.1.p	184.52	5	5	3.2912
RIB72	Cre02.g091700_4532.1.p	71.94	5	5	3.0372
TXC1	Cre11.g477950_4532.1.p	94.29	4	4	4.151
BIP2	Cre02.g080600_4532.1.p	75.24	3	6	4.2496
DAAK1	Cre01.g029750_4532.1.p	141.25	3	5	1.6642
HFO20	Cre13.g570000_4532.1.p	11.45	3	4	2.9745
DHC14	Cre09.g403800_4532.1.p	519.56	3	3	3.3479
FAP24	Cre02.g081050_4532.1.p	55.86	3	3	2.5289
FAP332	Cre02.g142687_4532.1.p	20.57	3	3	2.486
FAP148	Cre10.g434600_4532.1.p	203.41	3	3	2.3539
HSP70A	Cre08.g372100_4532.1.p	71.17	2	5	3.3658
LIP1	Cre09.g390615_4532.1.p	52.11	2	3	3.2967
VSP4	Cre09.g391801_4532.1.p	104.78	2	3	2.5674
rbcL	CreCp.g802313_4532.1.p	52.49	2	3	2.4885
CRB3	Cre03.g177600_4532.1.p	66.84	2	2	3.5428
PKHD1	Cre07.g340450_4532.1.p	512.51	2	2	3.3518
PHOT1	Cre03.g199000_4532.1.p	81.34	2	2	2.9532
METE1	Cre03.g180750_4532.1.p	94.66	2	2	2.5646
FAP199	Cre09.g399400_4532.1.p	126.46	2	2	2.423
CGL90	Cre11.g467632_4532.1.p	121.72	2	2	2.3432
POC14	Cre12.g559250_4532.1.p	29.5	2	2	2.3094
GAPC1	Cre12.g485150_4532.1.p	39.73	2	2	1.7637
GFY5	Cre17.g702950_4532.1.p	24.41	1	2	2.9848
RPS6	Cre09.g400664_4532.1.p	109.36	1	2	2.3942
FAP335	Cre01.g002550_4532.1.p	43.55	1	2	1.4846

Supplementary Table 3. **Unique peptides identified for MST3**. Related to Figure 5.

Peptide	Start residue	End Residue
FAVGDLTYTLVMQPR	792	806
TTTGDAVPDAVTSQPGNTFDVDAYR	4238	4262
IGDYLTEQR	4318	4326
GPPEVFVPLYGSYK	4442	4455
YAELVDTTGPTPPNTPFAITYDVK	4661	4684
DVSGNAAITQVR	4685	4696
GFLVGQLQR	5277	5285
NLQDLGGIGEDPVFDR	6370	6385
GVAAVAGGTALAK	6929	6941

Supplementary Table 4. Oligonucleotides used as primers for PCR validation of *C. reinhardtii* mutant strains. Related to Figure S4D-F.

Primer ID	Mutant strain validated	Sequence
F1	*mst3-1*	5′– GTCCGTGGTCGTGTCCTACG –3′
R1	*mst3-1*	5′– ACCAGCGTGTACGTGAGGTC –3′
F2	*mst3-2*	5′– GGTAGCCGTGGTGACATTAGC –3′
R2	*mst3-2*	5′– CAGCTCGTACTGCCCAAAGC –3′
F3	*mst3-3*	5′– AACACGCCCTTCGCCATCAC –3′
R3	*mst3-3*	5′– GCACAGAAGGCGTGAACCTG –3′
F4	*mst3-4*	5′– TTCGGTGTGGACCTGAAGGAC –3′
R4	*mst3-4*	5′– GGTGGCGCAGGTGGGCTG –3′
F5	*pkd2*	5′– CAAGCTGGACGAGATCATCG –3′
R5	*pkd2*	5′– GTTGGTGAGACCGACATTGC –3′
F6	*mst1*	5′– GAAGCACCTTGTCGCCTTG –3′
R6	*mst1*	5′– AAATAAGGTCGCTTTCGCCC –3′
F7	*sip*	5′– GAAAGTAGCGGTGAGTGACC –3′
R7	*sip*	5′– GGAGGAATGAGCACGCTAC –3′

References

[S1]. Gui, M., Wang, X., Dutcher, S.K., Brown, A., and Zhang, R. (2022). Ciliary central apparatus structure reveals mechanisms of microtubule patterning. Nat Struct Mol Biol 29, 483–492. 10.1038/s41594-022-00770-2.
